# Can changes in the distributions of resident birds in China over the past 50 years be attributed to climate change?

**DOI:** 10.1002/ece3.1513

**Published:** 2015-05-11

**Authors:** Jianguo Wu, Guobin Zhang

**Affiliations:** 1 The Center for Climate Change, Chinese Research Academy of Environment SciencesNo. 8 Da Yang Fang, Beiyuan, Anwai, Chaoyang District, Beijing, 100012, China; 2 Academy of Forest Inventory & Planning, State Forestry AdministrationNo. 18 Hepingli Dongjie, Eastern District, Beijing, 100714, China

**Keywords:** Attribution, biodiversity, climate change, detection, distribution, resident birds

## Abstract

The distributions of bird species have changed over the past 50 years in China. To evaluate whether the changes can be attributed to the changing climate, we analyzed the distributions of 20 subspecies of resident birds in relation to climate change. Long-term records of bird distributions, gray relational analysis, fuzzy-set classification techniques, and attribution methods were used. Among the 20 subspecies of resident birds, the northern limits of over half of the subspecies have shifted northward since the 1960s, and most changes have been related to the thermal index. Driven by climate change over the past 50 years, the suitable range and latitude or longitude of the distribution centers of certain birds have exhibited increased fluctuations. The northern boundaries of over half of the subspecies have shifted northward compared with those in the 1960s. The consistency between the observed and predicted changes in the range limits was quite high for some subspecies. The changes in the northern boundaries or the latitudes of the centers of distribution of nearly half of the subspecies can be attributed to climate change. The results suggest that climate change has affected the distributions of particular birds. The method used to attribute changes in bird distributions to climate change may also be effective for other animals.

## Introduction

Over the past 100 years, the global mean air temperature has increased by 0.85°C. The increase in the global mean surface temperature over 2081–2100 relative to 1986–2005 is projected to be in the range of 0.3–1.7°C (under Representative Concentration Pathways (RCPs), RCP2.6) or 2.6–4.8°C (under RCP8.5) (IPCC 2013). Climate change presents a challenge for biodiversity conservation (Thomas et al. 2004; Beever et al. 2011; Bellard et al. 2012; Moritz and Agudo 2013). Past climate change and other factors have already modified the distributions of species (Walther et al. 2005; Rubidge et al. 2011; Freeman and Freeman 2014). Detecting and explaining changes in species distributions is crucial for more accurate projecting the effects of climate change on the distributions of species in future (Hickling et al. 2006; Tingley et al. 2009; La Sorte and Jetz 2012; Ferrer-Paris et al. 2014; Virkkala et al. 2014). Among vertebrates, birds may be the most sensitive to climate change (Crick 2004; Lindström et al. 2012; McClure et al. 2012). The distributions of some bird species have changed in recent years (Norment et al. 1999; Huntley et al. 2006; Shoo et al. 2006; La Sorte and Thompson 2007). Detection and attribution of these changes is important for anticipating future distribution changes and extinctions of birds under climate warming (Davis et al. 1998; Thomas et al. 2006; Sekercioglu et al. 2008; Maggini et al. 2011; La Sorte and Jetz 2012).

Previous studies have found that some bird species have extended their ranges northward (Thomas and Lennon 1999; Hitch and Leberg 2007), including northern bird species (Norment et al. 1999; Brommer 2004; Crick 2004; Zuckerberg et al. 2009; Virkkala and Lehikoinen 2014). The elevational distributions of bird species have also changed (Sekercioglu et al. 2008; Zuckerberg et al. 2009), and some bird species have shifted toward higher elevations (Gregory et al. 2009; Popy et al. 2010; Maggini et al. 2011). However, compared with the total number of birds globally, the number of birds experiencing distributional changes remains relatively small.

Previous studies have analyzed the relationships between changes in bird distributions and climate factors (Venier et al. 1999; Forsman and Mönkkönen 2003; McClure et al. 2012). Some studies have focused on the effects of winter air temperature on bird distributions (Root 1988; Repasky 1991; Austain and Rehfisch 2005; Garamszegi et al. 2005; Butler et al. 2007; La Sorte and Thompson 2007; Maclean et al. 2008), and several studies have emphasized the effects of summer conditions on bird distributions (Zuckerberg et al. 2009; Lindström et al. 2012). Others have found that the interactions between latitude, longitude, temperature, and precipitation (Forsman and Mönkkönen 2003), dispersal capacity and temperature factors (Oswald and Arnold 2012) also influence changes in bird distributions. However, it is still uncertain for the most important factor affecting the distribution of some resident bird species (Braunisch et al. 2013).

If bird distribution changes are indeed mainly determined by climatic factors, the rapid climatic warming of the last three decades suggests that organisms should move their distribution poleward and toward higher altitudes (Parmesan 2006). However, many species have not exhibited a change in distribution in response to climate change (Parmesan and Yohe 2003; Parmesan 2006). Additionally, global climate has changed substantially over the past 100 years, but the changes to bird distributions have occurred in recent decades (Parmesan 1996; Thomas and Lennon 1999; Parmesan and Yohe 2003; Hickling et al. 2006). This pattern suggests that factors other than climate also influence the distribution limits of birds. Previous studies have found that the distribution of birds is influenced by many factors, including climate, habitat and species co-occurrence (Rubidge et al. 2011), topography, vegetation, and climate (Seoane et al. 2004), all of which are related to the effects of climate change. Additionally, species' ecological features can considerably alter the impacts of climate change (Brommer 2008; Reif and Flousek 2012). Land cover or habitat management, as well as climate (Delgado et al. 2009) and interactions among weather, urbanization, and supplemental food (Zuckerberg et al. 2011) all influence changes in bird distributions. Despite many studies about the relationship between bird distribution and environmental factors, there are insufficient data to detect and attribute the changes in bird distribution to climate change across regions.

In China, approximately 1300 bird species comprise 13% of the global bird diversity (Zheng 2011). Over time, the distributions of bird species have changed, and the changes have continued in recent years (Jiang and Wu 1988; Hu and Geng 1995; Dai et al. 1996; Zhang et al. 2001; Ma et al. 2008; Zhu and Li 2006; Ci et al. 2007; Gu et al. 2007; Zhang et al. 2006; Wang 2010). Some researchers have simply inferred that the changes are the result of climate change (Sun and Zhang 2000; Du et al. 2009), but whether the changes in bird distributions are attributable to climate change is inadequate.

The aim of this study was to detect changes in the distributions of nine resident birds over the past 50 years in China and to confirm that whether the changes can be attributed to climate change.

## Materials and Methods

### Bird distributions

**Figure 1:**
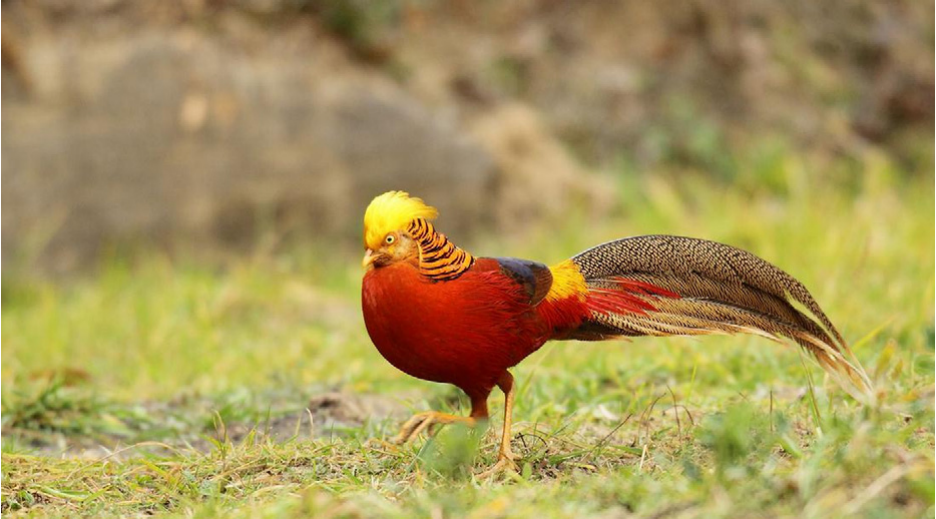
Golden pheasant (*Chrysolophus pictus*), which is endemic to China, with a broader distributional range.

Nine species and 20 subspecies of birds in China were selected for study: Black Baza (*Aviceda leuphotes leuphotes, Aviceda leuphotes wolfei,* and *Aviceda leuphotes syama*), Crested Goshawk (*Accipiter trivirgatus indicus* and *Accipiter trivirgatus formosae*), Shikra (*Accipiter badius cenchroides* and *Accipiter badius poliopsis*), Black Eagle (*Ictinaetus malayensis*), Crested Serpent Eagle (*Spilornis cheela burmanicus, Spilornis cheela ricketti, Spilornis cheela hoya,* and *Spilornis cheela rutherfordi*), Hodgson's Hawk Eagle (*Spizaetus nipalensis nipalensis* and *Spizaetus nipalensis orientalis*), Golden Pheasant (*Chrysolophus pictus*) (Fig.1), Brown Crake (*Amaurornis akool coccineipes*), and Spotted Dove (*Streptopelia chinensis chinensis, Streptopelia chinensis formosa, Streptopelia chinensis hainana,* and *Streptopelia chinensis tigrina*). These bird species were chosen for three reasons. First, these species are endangered in China (Zheng and Wang 1998), and it is important to conserve them as the climate changes. Second, complete point-distribution data are available for these bird species and subspecies; these data are crucial to analyzing the distributional changes. Although there are many bird species in China, fine-grain distributional data exist for only a few species. Third, in recent decades, many new distribution records have been found outside of the historical distribution boundaries of these bird species or subspecies (Jiang and Wu 1988; Hu and Geng 1995; Dai et al. 1996; Zhang et al. 2001; Ma et al. 2008; Zhu and Li 2006; Ci et al. 2007; Gu et al. 2007; Zhang et al. 2008; Wang 2010), and such records are also critical for identifying the distributional changes. Additionally, there have been many studies of the ecological traits of these birds (Gao 1996), and such studies are crucial for understanding the changes in the distributions of birds that are caused by climate change. In biological classification, a subspecies is either a taxonomic rank that is subordinate to species or a taxonomic unit in that rank. The differences among subspecies are usually less distinct than the differences among species. Because different subspecies of a given bird species are distributed in different climatic or ecological zones, and the distributions can be isolated and thus have very different climate, topographic, or other habitat conditions, we have analyzed changes in distributions at the subspecies level (Zheng 2011).

The distributions were determined from two groups of records: records before 1951 and records from 1951 to 2010. The data sources included national level distribution data and records from field investigations, samples reports, the China bird species distribution information database (http://www.birder.cn), the China animal database (http://www.zoology.csdb.cn), the annual report on bird watching in China in 2003–2010, Avifauna Sinica, avifaunal atlases and a geographical sketch of China as well as local or regional distribution data and records from censuses, investigation or samples of bird species in regional, provincial, district, county, and township level (Appendix S1). Additionally, the records of some bird distributions from natural area investigations and new records of bird distributions from observation are used (Appendix S1). To analyze bird distributional changes over specific time periods, the time series distribution records for each bird species were divided into decade time intervals: 1951 to 1960, 1961 to 1970, 1971 to 1980, 1981 to 1990, 1991 to 2000, and 2001 to 2010.

A large proportion of the bird survey data included different scales, and many records of prior distributions are provided as an approximate location or with a gazetteer (i.e., lacking the exact longitude and latitude of the location). Therefore, all of the bird distribution records were first geo-referenced to precise longitudes and latitudes. To improve the precision of the geo-referencing processes, we used an index of the Atlas of the People's Republic of China (The Restore Institute of Toponomy, Chinese State Bureau of Surveying and Mapping 1997) to interpolate the longitude and latitude records of every bird species distribution in China for each decade based on sightings or entries in the gazetteer index without coordinates. This index includes 33,211 gazetteer locations.

To reduce the bias resulting from temporal and spatial fluctuations in the samples, we removed the sites with extremely uncertain locations or with multiple entries that referenced the same specimen, and questionable distribution information was then cross-checked and corrected in the records by comparing similarities between climate, vegetation, and human activities. We also investigated temporal factors to verify and minimize data errors. We corrected bias in the presence or absence of bird distributions using a geographical sketch of China, China bird checklists (Zhang 1999; Cheng 1955, 1976, 1987; Cheng 1963) and a provincial bird checklist (see Appendix S1) that indicate bird species distributions throughout broad geopolitical, geographic or bioclimatic regions. We also used expert-drawn outlines of bird species distributions in Chinese avifaunal atlases and handbooks (Cheng 1959; MacKinnon et al. 2000), regardless of the resolution or false-positive rate of the species ecology, to reliably indicate a species absence outside of their known boundaries. Additionally, we used bird habitat preferences, elevation, and physiological tolerance limits as documented in the literature or by expert assessments coupled with fine-scale land cover, topography, and climate data to verify the presence/absence information (Gao 1996). This process generated a mean of approximately 1500 unique records with exact distribution information for per species or subspecies in the database. Because discrepancies occurred regarding the current distributions of particular birds across data sources, the distribution boundary was defined as *α*-hull (Burgman and Fox 2003). Because the data recorded only presence information, pseudo-absences were generated as in Zaniewski et al. (2002). Generating an absence in an area that is appropriate for a species is a risk (Zaniewski et al. 2002), although the general trends in bird distributions were not likely to be influenced.

### Climate change

Because there are direct or indirect relationships between bird distributions and macroclimate and microclimate, the mean annual temperature, mean temperature in January and July, sum of the cumulative temperatures above 0°C, minimum temperature in the coldest month, and maximum temperature in the warmest month were calculated. Additionally, the annual precipitation and Holdridge index (Holdridge 1967; Zhang 1993), including the mean annual biotemperature (BT) and annual potential evapotranspiration rate (PER), were selected (Appendix S2).

Climatic data for the last 60 years in China were provided by the climate center of the Chinese Administration of Meteorology as 17,625 grid cells with a resolution of 0.5°× 0.5°. Climatic variables were generated for each distribution point for each bird and each decade (see Appendix S2).

### Relationships between bird distribution and climate factors

To analyze how the bird distributions changed with climate, the coordinates for the northern, southern, western, and eastern limits and center coordinates of bird distributions were calculated. The limits were calculated based on the coordinates for the outermost 5% of occupied grid cells, which were considered to be the limit of species' ranges, whereas the center coordinates for the distribution were determined using the occupied grid cells. Changes in the range margin between any two decadal survey periods (e.g., 1981 to 1990 vs. 1991 to 2000) were estimated based on changes in the mean longitude and latitude of 5% of the most marginally occupied grid cells. Changes in center coordinate range between any two survey periods were estimated based on changes in the center coordinates of all occupied grid cells for the distribution of each bird species.

Conventional statistical methods that are frequently used to determine the relationship between independent and dependent factors include factor analysis and regression analysis. These analyses require a relationship of mutual influence between the variables, and the functional relationship can be elucidated only under the condition of large quantities of data that should conform to the normal distribution (Tsokos and Ramachandran 2009). Sometimes, the conditions for these statistical methods are not met. To overcome the shortage of data for regression and factor analysis, a multi-attribute method, gray relational analysis (GRA), was proposed (Deng 1987). Compared with regression and factor analysis, GRA has advantages, such as the ability to treat small samples, no normal distribution requirement, no independence requirement, and a small number of calculations (Deng 1987). GRA is a method for comparing different time series datasets, and it effectively reflects the relationship between the maximum extents of the time series variables (Deng 1987). Because of the errors that would be generated using conventional statistical analyses for a small sample size or data with a non-normal distribution (Deng 1987), we performed GRA to analyze the degree of gray incidence (DGI) of changes in bird distributions and climate factors using time series data of climate factor changes at the range limits or center coordinates of the distribution.

First, the time series data on the climate factors, range limits, and centers of distribution were normalized.

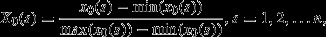
1a

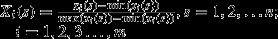
1b

where *x*_0_(*s*) and *x*_*i*_(*s*) are the original time series data on changes in the range limits or centers of distributions and the time series data on the i*th* climate factors, respectively; *X*_0_(*s*) and *X*_*i*_(*s*) are the normalized data points for changes in the range limits and centers of distributions and the i*th* climate factors, respectively; min (*x*_0_(*s*)), max (*x*_0_(*s*)), min (*x*_*i*_(*s*)), and max (*x*_*i*_(*s*)) are the minimum or maximum values for the range limits, centers of distributions, and climate factors, respectively; i and s are the i*th* climate variable and s*th* time interval, respectively.

Second, the absolute differences in the bird distributions and climate factors were calculated as follows:

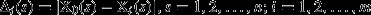
2

where Δ_*i*_(*s*) is the absolute distance between the normalized data sequence of the bird distributions and climate factors.

Third, the gray correlation coefficients of the changes in bird distributions and climate factors were calculated as follows:

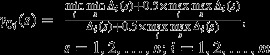
3

where *γ*_0*i*_(*s*) is the gray correlation coefficient of changes in bird distributions and climate factors; 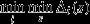 is the minimum value of the i*th* minimum value of the *Δ*_*i*_(*s*) sequence; 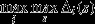 is the maximum value of the i*th* maximum value of the sequence *Δ*_*i*_(*s*); i and s are the i*th* climate variable and s*th* time interval, respectively; and 0*i* is the change in bird distributions and i*th* climate variable.

Finally, the degree of gray correlation change in bird distributions and climate factors was calculated as follows:

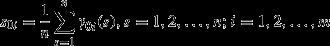
4

### Bird distributions driven by climate factors

Considering the risk of generating a false absence in an area with favorable bird habitat, the presence-only data on bird distributions were used in this study. The fuzzy envelope model can be used to predict the potential distribution of organisms using presence-only locality records and a set of environmental predictor variables (Robertson et al. 2004). Specifically, fuzzy models are appropriate for species distribution modeling because of their transparency and their ability to consider the uncertainty inherent to both biotic and abiotic variables (Van Broekhoven et al. 2006). Thus, fuzzy-set classification techniques were used to analyze the bird distributions influenced by climate factors over the past 50 years. First, 9 climate factors were used to describe past climate change (Appendix S2). The membership function of the climate factors was constructed based on the suitability of climate variables for bird survival and generation, and the membership of different climate factors shows their suitability for birds. The symmetric membership function of the Cauchy fuzzy distribution was used to describe the annual mean temperature, the mean temperatures in January and July, the sum of the cumulative temperatures above 0°C, the annual precipitation, BT and PER. The monotonically increasing function and monotonically decreasing function of the Cauchy fuzzy distribution were used to describe the minimum temperature in the coldest month and the maximum temperature in the warmest month, respectively (Appendix S3). The mean, minimum, and maximum of the suitable climate range of nine climate factors for different birds were computed based on the bird distributions and climate factors from 1951 to 1960 (i.e., the training dates) (Appendix S4). The parameter of the membership function of the different climate factors was optimally calculated by analyzing the membership of the most suitable point or nonsuitable point using the training dates (Appendix S5). Second, the suitability for birds of different climate factors for every grid cell for every year from 1961 to 2010 was calculated using the membership function according to climate factors. Third, the total membership of the factors was computed using the sum of the weighting coefficients multiplied by the membership of the climate factors at every grid cell from 1961 to 2010. The weighting coefficients of the climate factors were computed using the coefficient of variation of 33,211 location records of the climate variables (Appendix S6). Fourth, a multivariate set that represents the potential distribution of organisms was produced using the membership of different climate factors at every grid cell from 1961 to 2010. The localities with high values represent more favorable conditions for birds than those with low values.

The accuracy of the models was evaluated using the kappa-statistic (k). The presence and absence data determined by distribution records from 1951 to 1960 were used as the baseline, and the predicted and observed presence and absence records from 1961 to 1970, 1971 to 1980, 1981 to 1990, and 1990 to 2000 were used as an independent dataset for evaluating model performance (Robertson et al. 2004) (Appendix S7). The species distribution maps were created in ArcGIS (Vers. 9.3 for Windows, Esri Corp., 2008) according to the following criteria: membership of 0.61–1.00 = suitable for bird survival and membership of 0.00–0.60 = unsuitable for bird survival. The data were used in the point-coverage formats of ArcGIS. To reduce the bias, we used a maximum-likelihood approach based on a logistical regression to fit a species distribution model and estimate the historical probability of occurrence for each bird using the presence-only data (the Maxlike method, suggested by Royle et al. 2012).

### Agreement between observed and predicted distributions of birds

We defined the consistency index of the observed versus predicted changes in the range of bird based on the gray correlation grade of the observed and predicted changes. First, we calculated the observed and predicted changes in the distribution of birds, including their range limits and distribution center coordinates, using time series data for the observed and predicted distributions from 1961 to 2010. The northern, southern, western, and eastern limits were analyzed as the means of the coordinates of the outermost 5% of occupied grid cells, which were considered to be the range limits, and the center coordinates of the distribution were analyzed as the average coordinates for all of the occupied grid cells. We then analyzed the gray correlation coefficient between the observed and predicted ranges over the last 50 years using the time series data for the observed and predicted changes in bird distributions, which represented the consistency index of the observed versus predicted changes in the ranges of birds as follows.

First, the observed and predicted changes in bird distributions were normalized as follows:

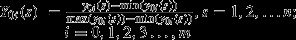
5a

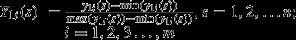
5b

where *y*_0*i*_(*s*) is the original sequence of the observed bird distributions, *y*_1*i*_(*s*) is the original sequence of the predicted bird distributions, *Y*_0*i*_(*s*) is the normalized data sequence of the observed bird distributions, and *Y*_1*i*_(*s*) is the normalized data sequence of the predicted bird distributions. The min (*y*_0*i*_(*s*)), max (*y*_0*i*_(*s*)), min (*y*_1*i*_(*s*)), and max (*y*_1*i*_(*s*)) are the minimum or maximum values of the original time series data of the observed and predicted changes in the range limits and the centers of distribution, respectively; and i and s are the i*th* range limits variable (northern, southern, western and eastern limits or centers of distribution) and s*th* time interval, respectively.

Second, the absolute difference between the observed and predicted bird distributions was calculated as follows:

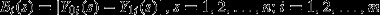
6

where *B*_*i*_(*s*) is the absolute distance between the normalized data sequences of observed and predicted distributions, *Y*_0*i*_(*s*) is the normalized data sequence of the observed distribution, and *Y*_1*i*_(*s*) is the normalized data sequence of predicted distribution.

Third, the gray correlation coefficients of the observed and predicted distribution changes were calculated as follows:

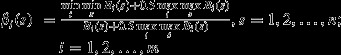
7

where *β*_*i*_(*s*) is the gray correlation coefficient of the observed and predicted changes; 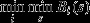 is the minimum value of the i minimum value of the B_i_(*s*) sequence; 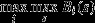 is the maximum value of the i maximum value of the sequence of B_i_(*s*); i and s are the i*th* distribution variables and s*th* time intervals, respectively.

Fourth, the consistency index of the observed and predicted changes in a bird's distribution was then calculated as follows:

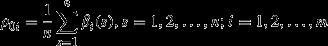
8

### The attribution of changes in bird distributions

The observed changes in the distribution of bird species cannot be attributed to past climate change if 1) there are no changes in the observed or predicted distributions based on climate factors, 2) there is no consistency between the observed and predicted changes in distribution, or 3) there is a poor relationship between the observed changes in bird distribution and climate change. Therefore, the degree of attribution to climate change of the changes in bird distribution (*A*_*ij*_) was defined as a function of the observed changes in bird distribution (*O*_*ij*_), correlation between the climatic factors and changes in distribution (*R*_*ij*_), predicted changes in the distribution (*S*_*ij*_), and consistency between the observed and predicted changes in distribution (*C*_*ij*_). Mathematically, this relationship is expressed as follows:

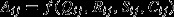
9

we assume that *O*_*ij*_, *R*_*ij*_, *S*_*ij*_, and *C*_*ij*_ are of equal importance, and all are required to determine *A*_*ij*_; thus, we redefine eq. 9 as follows: 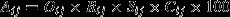
10

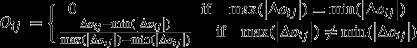
11

where *Δo*_*ij*_,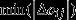, and 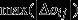 are the observed change in the range limit and minimum and maximum value of its absolute value, respectively.

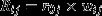
12

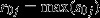
13

where *s*_0*i*_ is calculated using eq. 4 and max (*s*_0*j*_) is the maximum value of *s*_0*j*_.

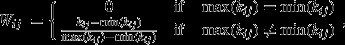
14

where *w*_*ij*_ is the change in the climate factor and *k*_*ij*_, min (*k*_*ij*_), and max (*k*_*ij*_) are the simple correlation between the change in climate factors and time and its minimum and maximum value, respectively.

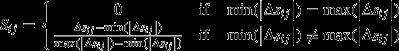
15where *Δs*_*ij*_, 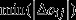 and 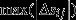 is the predicted change in the range limits and minimum and maximum value of its absolute value, respectively.

*C*_*ij*_ equal to *ρ*_0*j*_, which is calculated using eq. 8.

Higher values of *A*_*ij*_ indicate that the changes in bird distributions are better attributed to climate change, and if *A*_*ij*_ is less than or equal 0, the changes in bird distributions cannot be attributed to climate change.

## Results

### Changes in bird distributions

**Figure 2:**
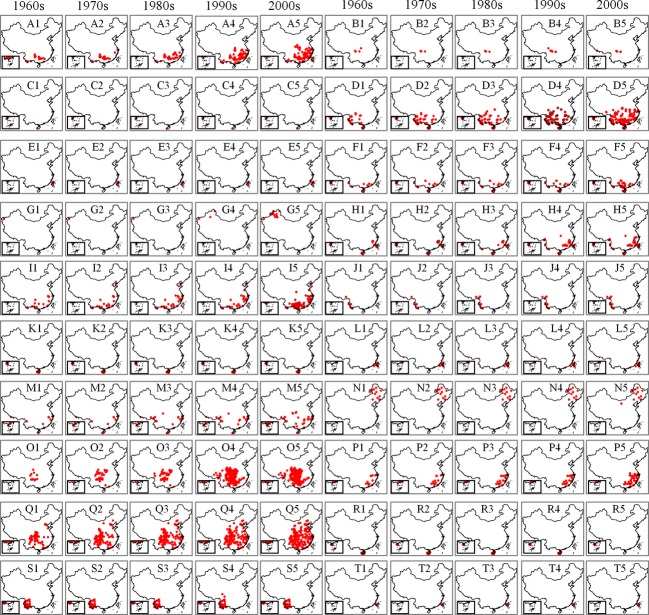
The observed distribution of birds in the 1960s, the 1970s, the 1980s, the 1990s and the 2000s. Notes: A, B, C, D, E, F, G, H, I, J, K, L, M, N, O, P, Q, R, S, and T represent *Aviceda leuphotes leuphotes, Aviceda leuphotes wolfei, Aviceda leuphotes syama, Accipiter trivirgatus indicus*, *Accipiter trivirgatus formosae, Accipiter badius cenchroides, Accipiter badius poliopsis, Ictinaetus malayensis, Spilornis cheela burmanicus, Spilornis cheela ricketti, Spilornis cheela hoya, Spilornis cheela rutherfordi, Spizaetus nipalensis nipalensis, Spizaetus nipalensis orientalis, Chrysolophus pictus, Amaurornis akool coccineipes, Streptopelia chinensis chinensis, Streptopelia chinensis formosa, Streptopelia chinensis hainana, Streptopelia chinensis tigrina, r*espectively.

Bird distributions vary by decade and species or subspecies (Fig.2). Among the 20 subspecies, the latitude of the centers of distribution for seven subspecies of birds, the southern limit of four subspecies, the northern limit of 12 subspecies, the longitude of the centers of distribution of nine subspecies*,* and the western and eastern limits of eight subspecies experienced obvious changes in the 1970s compared with the 1960s (Fig.2).

Compared to the 1960s data, the northern limit of 11 subspecies shifted northward, the southern limit of four subspecies shifted southward, the western limit of eight subspecies shifted westward, and the eastern limit of seven subspecies shifted eastward. The centers of distribution shifted northward for seven species, eastward for six species, and westward for three species (Fig.2).

### Relationships between changes in bird distributions and climate factors

**Figure 3:**
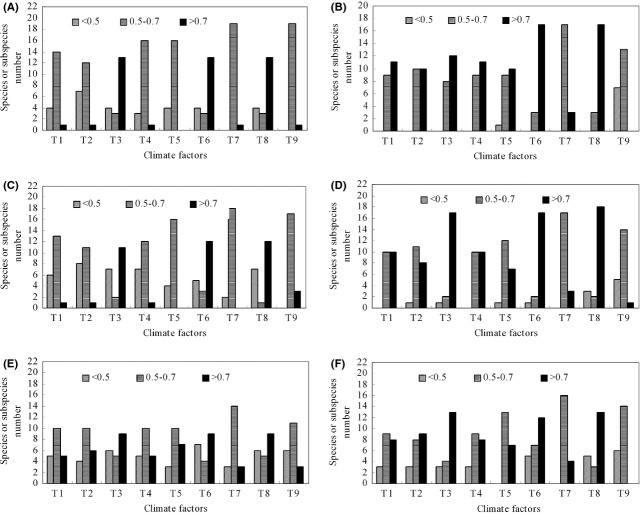
The number of species or subspecies with different degrees of gray incidence of observed changes at southern (A), northern (B), eastern (C), and western (D) distribution boundaries, and latitude (E) and longitude (F) of the distribution centers of birds with different climatic factors. Notes: T1, T2, T3, T4, T5, T6, T7, T8, and T9 represent mean annual air temperature, mean air temperature in January, mean air temperature in July, the highest temperature in the warmest month, the lowest temperature in the coldest month, sums of cumulative temperature above 0°C,annual precipitation, BT and PER, respectively.

The degree of gray incidence (DGI) relating changes in the southern and northern limits to climate factors varied (Fig.3; Appendix S8). The southern limit change of 14 subspecies was mainly related to changes in the thermal index, whereas that of two subspecies was mainly related to changes in PER; the southern limit change of four subspecies was mainly related to changes in precipitation. Changes in the northern limit of 18 subspecies were mainly related to changes in temperature-related indices, and those of two other subspecies were mainly related to changes in PER or precipitation.

The DGI of changes to the eastern or western limits as related to climate factors varied (Fig.3; Appendix S9). The eastern limit changes of 12 subspecies of birds were mainly related to changes in temperature-related indices, whereas those of five subspecies were mainly related to changes in precipitation; the eastern limit changes of three subspecies were mainly related to changes in PER. The changes to the western limits of 18 subspecies were mainly related to increases in the temperature-related indices, whereas those of other subspecies were mainly related to changes in PER or precipitation.

The longitudes of the centers of distribution for 10 subspecies were mainly related to changes in temperature-related indices, whereas those of six subspecies were mainly related to changes in precipitation; those longitudes of four subspecies were mainly related to changes in PRE (Fig.3; Appendix S10). The latitudes of the centers of distribution for 13 subspecies were mainly related to changes in temperature-related indices, whereas those of other subspecies were mainly related to changes in PER or precipitation.

### Bird distributions response to climate factors

**Figure 4:**
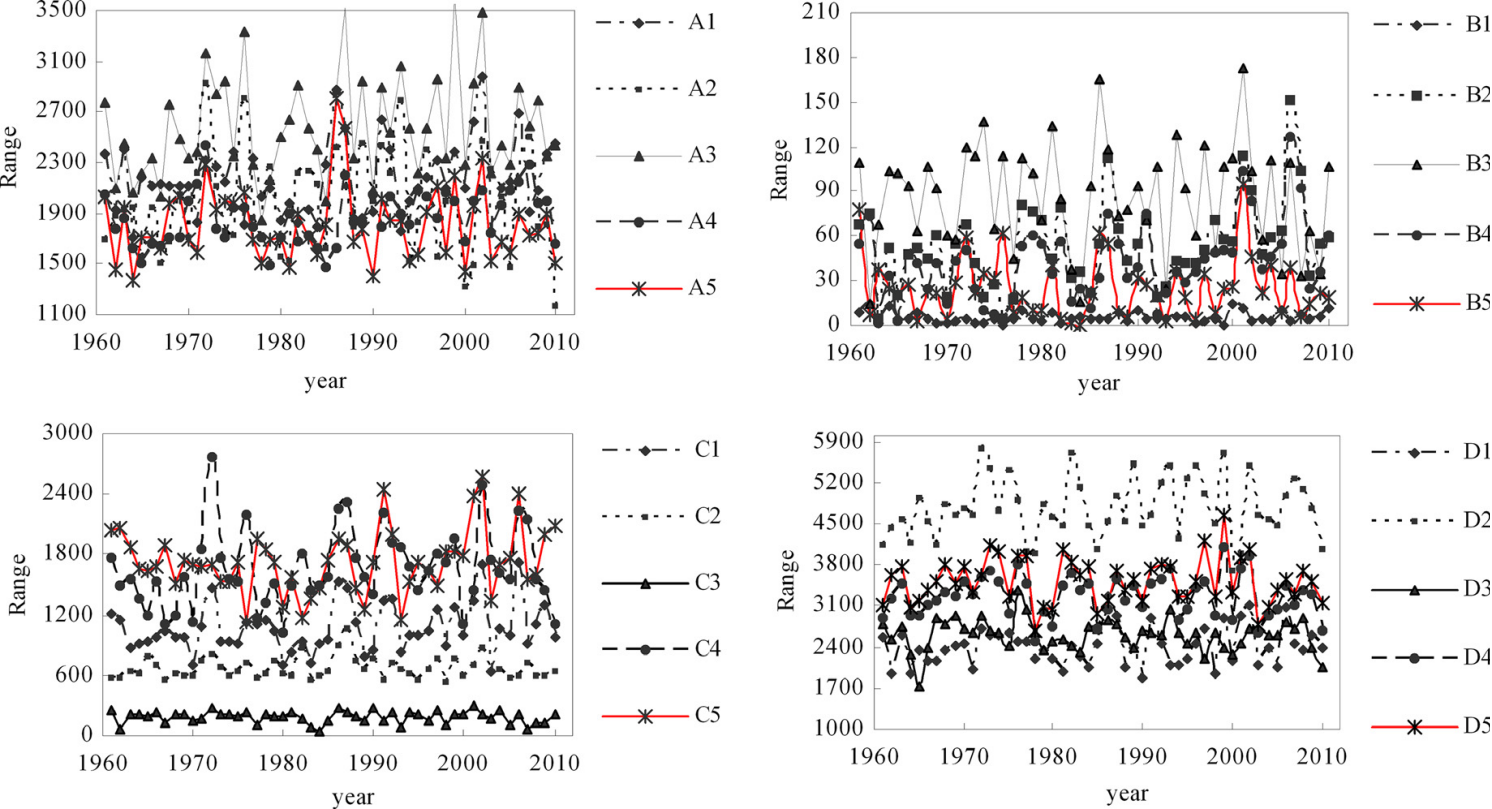
The range change of the predicted distribution of birds driven by climate factors. Note: A1, A2, A3, A4, A5, B1, B2, B3, B4, B5, C1, C2, C3, C4, C5, D1, D2, D3, D4, D5 represent *Aviceda leuphotes leuphotes, Aviceda leuphotes wolfei, Accipiter trivirgatus indicus, Accipiter badius poliopsis, Ictinaetus malayensis, Aviceda leuphotes syama, Streptopelia chinensis formosa, Accipiter trivirgatus formosae, Spilornis cheela hoya, Streptopelia chinensis tigrina, Accipiter badius cenchroides, Spilornis cheela ricketti, Spilornis cheela rutherfordi, Streptopelia chinensis hainana, Amaurornis akool coccineipes, Spilornis cheela burmanicus, Spizaetus nipalensis nipalensis, Spizaetus nipalensis orientalis, Chrysolophus pictus, Streptopelia chinensis chinensis,* respectively.

**Figure 5:**
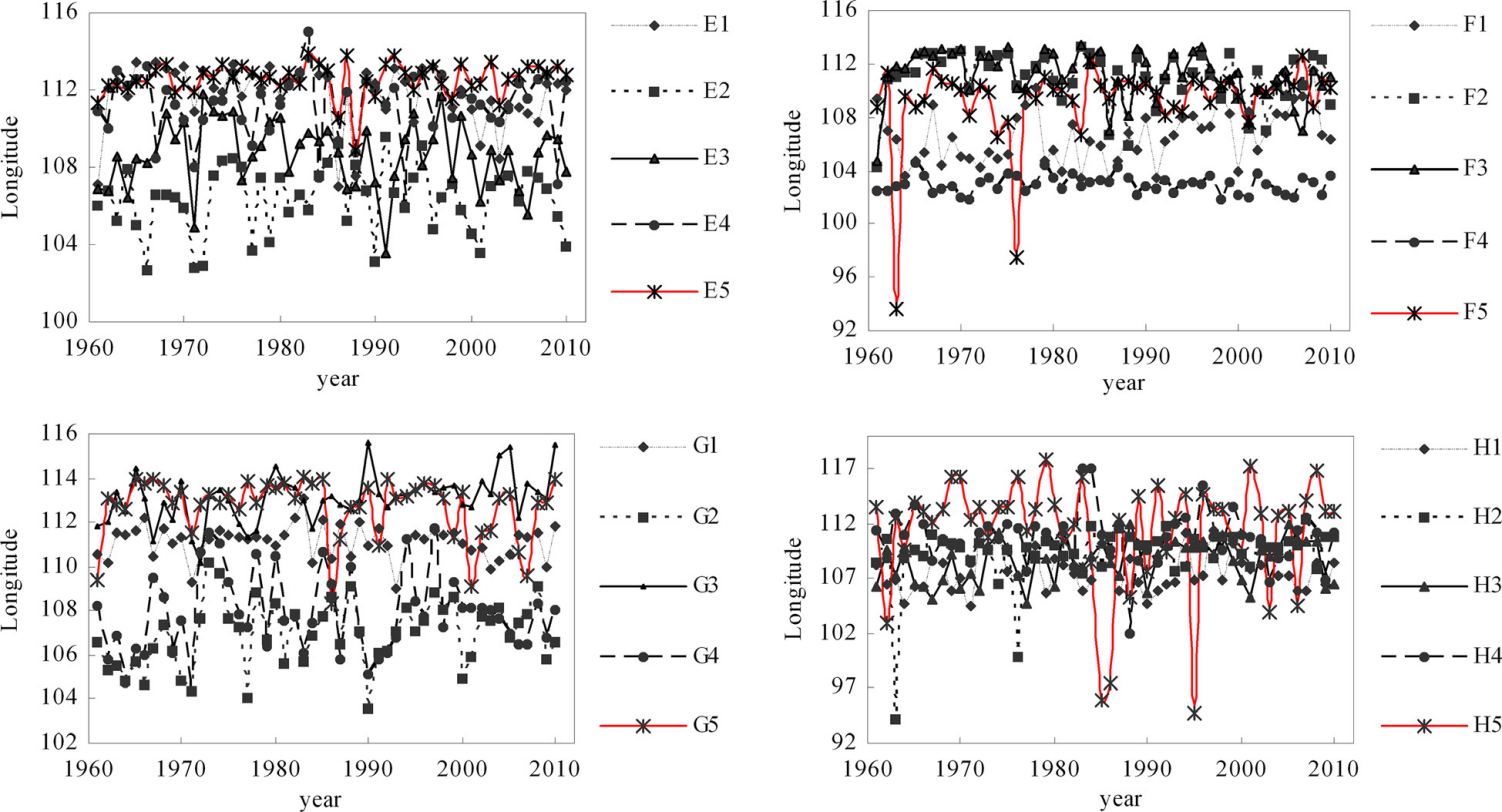
The longitude change of predicted distribution centers of birds driven by climate factors. Note: E1, E2, E3, E4, E5, F1, F2, F3, F4, F5, G1, G2, G3, G4, G5, H1, H2, H3, H4, H5 represent *Aviceda leuphotes leuphotes, Aviceda leuphotes wolfei, Accipiter trivirgatus indicus, Accipiter trivirgatus formosae, Accipiter badius cenchroides, Accipiter badius poliopsis, Ictinaetus malayensis, Spilornis cheela burmanicus, Spilornis cheela ricketti, Spilornis cheela hoya, Spilornis cheela rutherfordi, Spizaetus nipalensis nipalensis, Spizaetus nipalensis orientalis, Chrysolophus pictus, Amaurornis akool coccineipes, Streptopelia chinensis chinensis, Streptopelia chinensis formosa, Streptopelia chinensis hainana, Streptopelia chinensis tigrina, Aviceda leuphotes syama, r*espectively.

**Figure 6:**
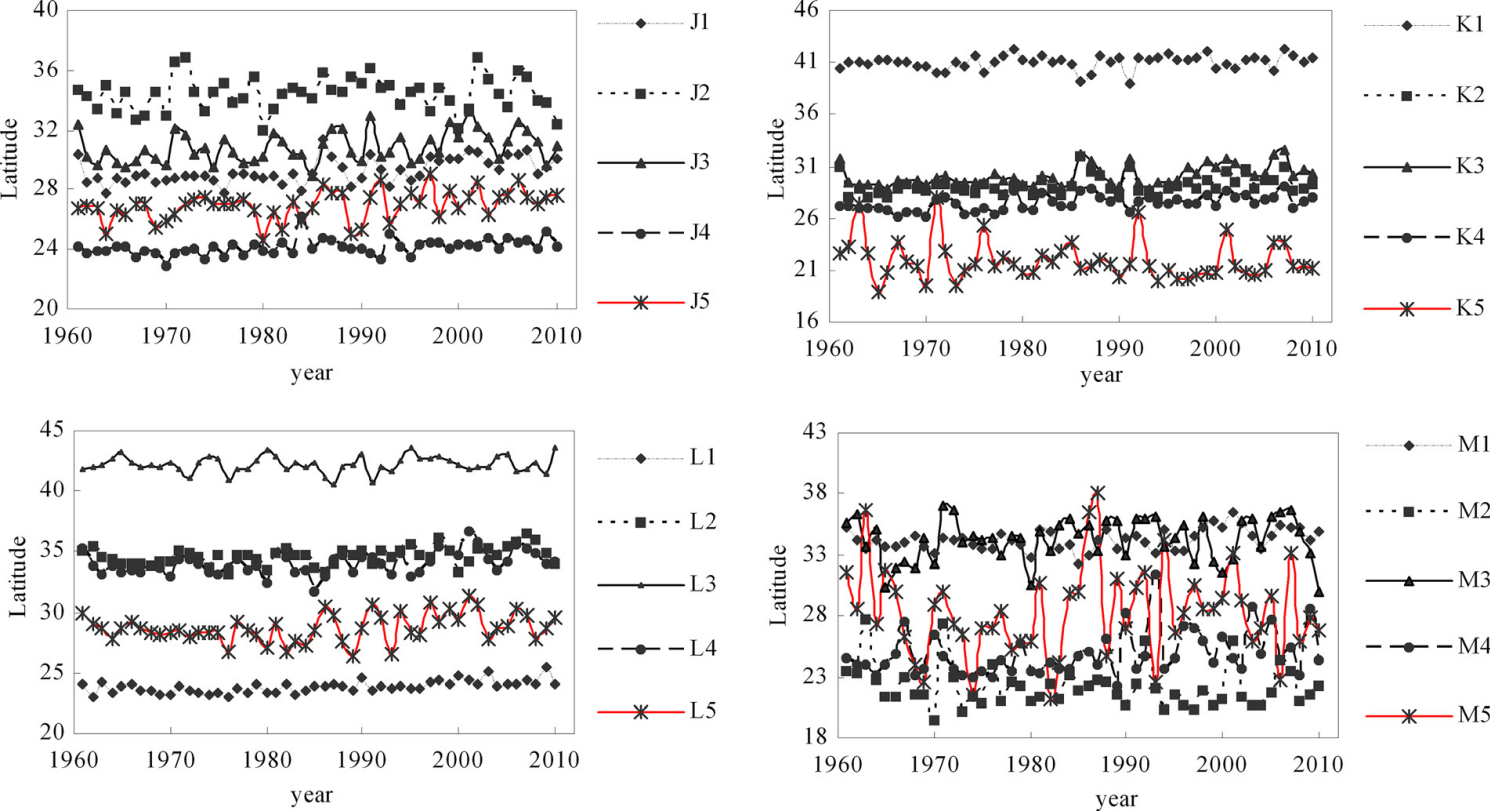
The latitude changes of predicted distribution centers of birds driven by climate factors. Note: J1, J2, J3, J4, J5, K1, K2, K3, K4, K5, L1, L2, L3, L4, L5, M1, M2, M3, M4, M5 represent *Aviceda leuphotes leuphotes, Aviceda leuphotes wolfei, Accipiter trivirgatus indicus, Accipiter trivirgatus formosae, Accipiter badius cenchroides, Accipiter badius poliopsis, Ictinaetus malayensis, Spilornis cheela burmanicus, Spilornis cheela ricketti, Spilornis cheela hoya, Spilornis cheela rutherfordi, Spizaetus nipalensis nipalensis, Spizaetus nipalensis orientalis, Chrysolophus pictus, Amaurornis akool coccineipes,S treptopelia chinensis chinensis, Streptopelia chinensis formosa, Streptopelia chinensis hainana, Streptopelia chinensis tigrina, Aviceda leuphotes syama,* respectively.

With climate-driven changes over past 50 years, the suitable distribution range of nine subspecies of birds appeared to increase with great fluctuation, whereas the distributions of other bird species or subspecies appeared to exhibit no change with fluctuation or to increase then decrease (Fig.4). Additionally, the longitude of the centers of distribution of eight subspecies appeared to increase with fluctuations, whereas that of other birds appeared to decrease with fluctuation, or to exhibit no change (Fig.5), and the latitude of the centers of distribution of 10 subspecies appeared to increase with fluctuations, whereas that of other birds appeared to exhibit no change with fluctuation, or to increase then decrease (Fig.6).

**Figure 7:**
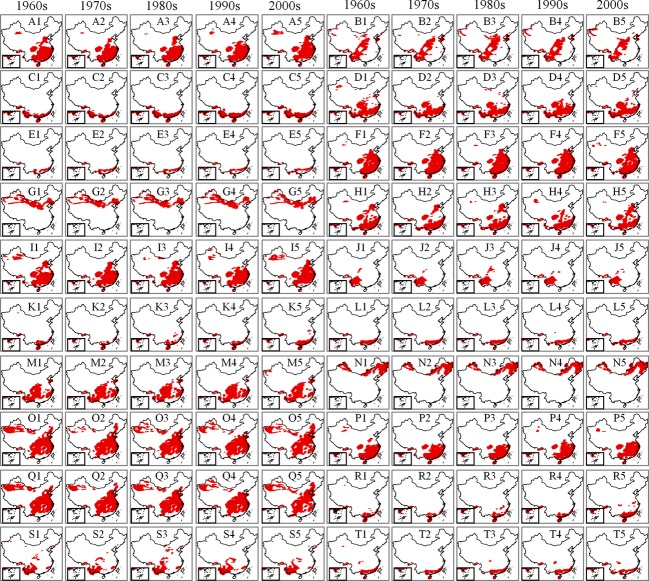
The predicted distribution of birds in the 1960s, the 1970s, the 1980s, the 1990s, and the 2000s driven by climate factors. Notes: A, B, C, D, E, F, G, H, I, J, K, L, M, N, O, P, Q, R, S, and T represent *Aviceda leuphotes leuphotes, Aviceda leuphotes wolfei, Aviceda leuphotes syama, Accipiter trivirgatus indicus*, *Accipiter trivirgatus formosae, Accipiter badius cenchroides, Accipiter badius poliopsis, Ictinaetus malayensis, Spilornis cheela burmanicus, Spilornis cheela ricketti, Spilornis cheela hoya, Spilornis cheela rutherfordi, Spizaetus nipalensis nipalensis, Spizaetus nipalensis orientalis, Chrysolophus pictus, Amaurornis akool coccineipes, Streptopelia chinensis chinensis, Streptopelia chinensis formosa, Streptopelia chinensis hainana, Streptopelia chinensis tigrina, r*espectively.

Compared with bird distributions in the 1960s, the northern boundaries of the distributions of 10 subspecies have shifted northward, the southern boundaries of two subspecies have shifted northward, the western boundaries of five subspecies have shifted westward, the western boundaries of two subspecies have shifted eastward, the eastern boundaries of eight subspecies have shifted eastward, the centers of distribution of seven subspecies have shifted northward, the centers of distribution of two subspecies have shifted southward, the centers of distribution of six subspecies have shifted eastward, and the center of distribution of one subspecies has shifted westward (Fig.7).

### The consistency between observed and predicted distributions of birds

The consistency index of observed changes and predicted changes in the ranges of different birds over the past 50 years varies among the subspecies. The consistency index of the latitudes of the centers of distribution of five subspecies is above 0.7, that of the southern boundaries of four subspecies is above 0.7, and that of northern boundaries of six subspecies is above 0.7 (Table1). The consistency index of the longitudes of the centers of distribution of four subspecies is above 0.7, that of the eastern boundaries of six subspecies is above 0.7, and that of the western boundary of four subspecies is above 0.7 (Table1). These findings revealed the high consistency between the observed changes and the predicted changes in the ranges of certain birds over the past 50 years.

**Table 1:** The consistency index of the observed and predicted changes in bird distribution

Species or subspecies of birds	C–W	S	N	C–J	W	E
*Aviceda leuphotes leuphotes*	0.59	0.60	0.77	0.66	0.70	0.60
*Aviceda leuphotes wolfei*	0.72	0.67	0.52	0.66	0.75	0.80
*Aviceda leuphotes syama*	0.52	0.55	0.56	0.55	0.54	0.60
*Accipiter trivirgatus indicus*	0.52	0.67	0.66	0.72	0.76	0.61
*Accipiter trivirgatus formosae*	0.72	0.58	0.58	0.63	0.65	0.54
*Accipiter badius cenchroides*	0.80	0.00	0.62	0.70	0.60	0.52
*Accipiter badius poliopsis*	0.62	0.60	0.70	0.62	0.57	0.64
*Ictinaetus malayensis*	0.71	0.74	0.75	0.57	0.70	0.78
*Spilornis cheela burmanicus*	0.65	0.89	0.58	0.60	0.63	0.56
*Spilornis cheela ricketti*	0.66	0.74	0.69	0.71	0.49	0.60
*Spilornis cheela hoya*	0.54	0.72	0.56	0.56	0.48	0.65
*Spilornis cheela rutherfordi*	0.64	0.65	0.75	0.58	0.53	0.61
*Spizaetus nipalensis nipalensis*	0.66	0.60	0.67	0.65	0.61	0.62
*Spizaetus nipalensis orientalis*	0.55	0.65	0.47	0.76	0.67	0.47
*Chrysolophus pictu*	0.59	0.67	0.71	0.65	0.77	0.65
*Amaurornis akool coccineipes*	0.64	0.63	0.70	0.59	0.58	0.80
*Streptopelia chinensis chinensis*	0.72	0.67	0.61	0.66	0.48	0.58
*Streptopelia chinensis formosa*	0.57	0.64	0.66	0.62	0.59	0.71
*Streptopelia chinensis hainana*	0.56	0.00	0.64	0.64	0.87	0.64
*Streptopelia chinensis tigrina*	0.55	0.55	0.56	0.57	0.53	0.51

Notes: C–J, C–W stand for the longitude and latitude of the centers of distribution, respectively; S, N, W, and E stand for the southern boundary, northern boundary, western boundary and eastern boundary of bird distributions, respectively.

### The attribution of bird distribution changes

The extent to which changes in the latitudes of the centers of distribution, the southern and northern boundaries, the longitudes of the centers of distribution, and the western and eastern boundaries of the distributions of different birds can be attributed to climate change is variable (Table2). The latitudinal changes in the centers of distribution are greater for eight subspecies of birds; the northern boundary changes are greater for nine subspecies; the longitudinal changes in the centers of distribution are greater for three subspecies; the western boundary changes are greater for one subspecies; and the eastern boundary changes are greater for six subspecies (Table2). These changes in bird distributions can be attributed to climate change.

**Table 2:** The degree of attribution of observed changes in bird distribution to climate change

Species or subspecies of birds	C–W	S	N	C–J	W	E
*Aviceda leuphotes leuphotes*	4.84	0.00	8.95	−1.24	0.00	0.19
*Aviceda leuphotes wolfei*	−0.50	0.00	−0.37	−0.46	0.00	−0.21
*Aviceda leuphotes syama*	0.00	0.00	0.00	0.00	0.00	0.00
*Accipiter trivirgatus indicus*	2.65	0.00	1.79	0.60	0.00	8.64
*Accipiter trivirgatus formosae*	0.27	0.00	−0.66	0.00	0.00	0.00
*Accipiter badius cenchroides*	9.93	0.00	0.00	−0.09	0.00	0.00
*Accipiter badius poliopsis*	3.63	0.00	3.26	3.23	0.00	27.99
*Ictinaetus malayensis*	2.80	0.00	5.04	0.05	0.52	0.00
*Spilornis cheela burmanicus*	0.00	−3.75	28.16	0.00	0.13	1.44
*Spilornis cheela ricketti*	5.02	0.00	0.51	0.04	0.00	0.00
*Spilornis cheela hoya*	0.00	0.00	0.00	0.00	0.00	0.00
*Spilornis cheela rutherfordi*	0.00	0.00	0.00	0.00	0.00	0.00
*Spizaetus nipalensis nipalensis*	0.00	−0.11	6.93	−0.17	0.00	0.99
*Spizaetus nipalensis orientalis*	0.00	0.00	0.00	0.00	0.00	0.00
*Chrysolophus pictu*	1.06	−3.17	1.69	1.09	0.40	15.07
*Amaurornis akool coccineipes*	1.79	0.00	2.41	0.00	0.00	0.02
*Streptopelia chinensis chinensis*	−0.33	0.00	1.74	1.47	0.00	5.34
*Streptopelia chinensis formosa*	0.00	−0.62	−0.05	0.00	10.44	3.55
*Streptopelia chinensis hainana*	0.00	0.00	0.00	0.00	0.00	0.00
*Streptopelia chinensis tigrina*	0.00	0.00	0.00	0.05	0.00	0.00

Notes: C–J, C–W stand for the longitude and latitude of the centers of distribution, respectively; S, N, W, and E stand for the southern boundary, northern boundary, western boundary and eastern boundary of bird distributions, respectively.

## Discussion

### Changes in bird distribution

The results showed that the distributions of over half of the 20 subspecies have clearly expanded northward since the 1970s, and the northern range limits of the birds have also shifted northward compared with the 1960s (Fig.3). These results agreed with those of Norment et al. (1999), Brommer (2004), and Crick (2004). Changes in other directions were also detected in our study. These results are consistent with an increasing number of reports that have documented other types of range shifts, such as east–west shifts across longitudes or even shifts toward tropical latitudes and lower elevations (Lenoir and Svenning 2015). These results imply that directions beyond the northern limit should be considered when evaluating distribution changes of species. Furthermore, linear changes in bird distribution over time were not observed in our study. This may result from spatial heterogeneity among the environmental factors (Newton 2003; Shoo et al. 2006; Tingley et al. 2012).

In our study, the distribution records of bird species or subspecies over the past 50 years were used to detect changes in the distribution of bird species or subspecies. However, documenting changes requires a reliable representation of current and past distributions (Tingley and Beissinger 2009). There are many bird species in China, but fine-grained distribution information is available for only a limited number of species. Therefore, we selected endangered bird species or subspecies with well-documented past distributions for which records of new distributions outside of their distribution boundaries in recent decades are available. If we had chosen bird species for which the past distributions are not well known and found a change in their distributions, then the observed changes might have been caused by bias from a poor sample effort (Tingley and Beissinger 2009; Kujala et al. 2013). In addition, if we had chosen bird species for which past distribution records are complete and without any information about new distribution records outside the historical distribution boundaries or without any information about absent distribution records inside the historical distribution boundaries, it would provide insufficient evidence to identify species distribution change from this documenting information (Tingley and Beissinger 2009). Furthermore, if we do not indiscriminately detect and attribute the changes in all bird species distribution to climate change, then insufficient data about the bird species' distribution changes will result in biases or errors, and the conclusions will be more unreliable.

Some studies have found that a common set of species-level traits explained differential responses among the species to climate change; an example is that species with smaller clutch sizes and stricter diets exhibited greater northward shifts, whereas species with larger clutch sizes and stricter diets exhibited increased elevational shifts (Auer and King 2014), and the changes in the mean weighted latitude of the density of 94 bird species in Finland, northern Europe, were significantly stronger in the northern species compared with the southern species using data covering a north–south gradient of over 1000 km from the 1970s to the 2010s (Virkkala and Lehikoinen 2014). Our results showed that the changes in distributions vary by subspecies or species. The changes may be related to ecological and life-history traits of the different bird species or subspecies (Auer et al. 2014).

Previous studies have detected distribution shifts by determining the difference between two bird atlas surveys (Väisänen 1998) or using repeated mapping surveys of species (Thomas and Lennon 1999). The approach used here correctly identifies the observed changes in the range limit or range size but does not necessarily account for potential biases in the sample; additionally, temporal changes in the distribution of birds based on repeated mapping surveys may be inflated by changes in the survey methodology (Kujala et al. 2013). To estimate potential changes in the distribution of Amazon parrots, Ferrer-Paris et al. (2014) combined bird survey data with historical distribution records and used a maximum-likelihood method to fit a species-distribution model and estimate a historical maximum probability of occurrence for each species. Such an approach takes advantage of limited available data to detect a high probability of change, even for rare and nonuniformly distributed species. However, the technique is presently limited to species that adhere to the strong assumptions required for maximum-likelihood estimations with the presence-only data (Ferrer-Paris et al. 2014). In our study, we used the distribution records of birds in different periods over 50 years to detect range changes, and used different methods to reduce the bias from poor sample efforts in some regions, or incorrect records, coarse resolution, or no information on species occurrence. The change in the trend of different birds species or subspecies can been effectively identified, and some sampling bias may be decreased when we checked and corrected some biases in the presence and absence data regarding bird distributions using a geographical sketch of China and bird checklists for China as a whole as well as for individual provinces, or using Chinese avifaunal atlases and handbooks even if they are coarse-grained and suffer high false-positive rates that vary with species ecology. Because the bird distributions did not shift as a whole and continuously or linearly but shifted with spatial heterogeneity, we detected changes in species distributions by identifying “pioneer” changes in the locations of distribution boundaries but not all distributional ranges of bird species or subspecies. Furthermore, the trends identified in the distribution changes for each bird subspecies depended on differences of occurrence, presence and absence, and distribution boundaries of the birds over time within a broad area (e.g., geopolitical, geographic, or bioclimatic regions) as indicated by a geographical sketch of China, China bird checklists, provincial birds checklists and expert Chinese avifaunal atlases and handbooks, which indicate absences of birds outside their boundaries or the absences over large areas; therefore, we detected distributional changes of bird species by comparing the differences of the new occurrence, presence and absence, or distribution boundaries records of the birds species or subspecies within broad geographic areas in different time periods (Tingley and Beissinger 2009). These efforts reduced the sampling bias.

### The relationship between distributional changes of birds and climate factors

Climate factors are crucial in determining the distributions of birds (Parmesan 1996; Venier et al. 1999; Forsman and Mönkkönen 2003); changes in bird distributions may be related to different climate factors (McClure et al. 2012). To detect and attribute the distributional changes of birds to climate change over the past years, the analysis of the relationship between the changes in the distribution of birds and climatic factors is required. Some studies have found that past winter temperature changes affect the distributions of birds (Root 1988; Repasky 1991; Garamszegi et al. 2005; Butler et al. 2007; La Sorte and Thompson 2007; La Sorte and Jetz 2012), and others have found that summer conditions drive the distribution of birds (Jiguet et al. 2003; Zuckerberg et al. 2009; Lindström et al. 2012). In our study, both the changes in the characteristics of observed different climate factors and observed changes in distribution have differed over the past 50 years. Therefore, it is important to detect which climate factor change is most important concerning changes in species distribution when we identify whether changes in species distribution can be attributed to climate change over the past years. Our results showed that the changing trends in the northern boundaries of the majority of bird subspecies mimics trends of temperature change such that the northern boundaries of some birds have shifted northward with increasing temperatures, and the southern, western, or eastern boundaries of a few species have shifted with increasing temperature; these shifts have intensified over the past 50 years in China (Fig.3). Our results also showed the centers of distribution have shifted in response to temperature factors because of the direct impacts of climatic warming on heat stress in endothermic species (Oswald and Arnold 2012). Additionally, some studies have emphasized the effect of precipitation changes on the distribution of species (Tingley et al. 2012). The changes in the distribution of some birds in our study are related to precipitation but only slightly. These changes are unlikely to reflect strong effects of precipitation changes because the range changes were small and trends in annual precipitation fluctuated through time.

Our results also showed that the relationship between distribution changes and climate factors depends on the species. This relationship can be affected by the bird's original distribution, differences in climate factors, or the interaction of climate factors and other factors, such as habitat (Rubidge et al. 2011), habitat management (Delgado et al. 2009), topography and vegetation (Windstorm et al. 2012), the link between weather and food (Zuckerberg et al. 2011), or species' ecological features (Brommer 2008; Reif and Flousek 2012). Thus, the changes in the distributions of birds are influenced by various factors.

GRA is used to identify the relationship between a reference sequence and a comparative sequence by calculating the gray relational grade. This technique is an appropriate method for measuring the similarities or differences among observations to analyze the relational structure (Deng 1987). We identify which climate factor changes are the most important concerning changes in the distribution of bird species using GRA.

We used the *α*-hull-defining distribution boundary. Although convex hulls (minimum convex polygons) are an internationally accepted standard method for estimating species' ranges, particularly when the presence-only data are the only type of spatially explicit data available, the method excludes cases of vagrancy and disjunctions within the overall distributions of taxa (Burgman and Fox 2003). This method assesses areas and trends in occupied habitats, and it is important for determining the conservation status of a species (Burgman and Fox 2003). A weakness of this method is that the constraint of convexity yields a hull with a very coarse outer resolution, resulting in a substantial overestimation of the range, particularly for irregularly shaped species ranges (Burgman and Fox 2003). The bias is influenced by the underlying shape of the species habitat, the magnitude of locational errors, and the spatiotemporal distribution of the sampling effort. Some of these errors may be reduced through the application of *α*-hulls, which are generalizations of convex hulls that provide an explicit means for excluding discontinuities within a species range (Burgman and Fox 2003). Convex hulls exhibit larger biases than *α*-hulls (Burgman and Fox 2003).

### Bird distribution in response to climate factors

The current study suggests that the ranges and distribution center coordinates of particular bird species have primarily shifted northward or westward and were driven by climate factors (with fluctuation). However, the fuzzy-set classification techniques used to predict changes of bird distribution assumed that a type of equilibrium occurred in the environmental niche. The plausibility of this assumption depends on the model scale and species dispersal ability and history (Araújo and Pearson 2005). Birds are strongly dependent on climate because of their specific life-history traits, including breeding, diet, and other behaviors (Jiménez-Valverde et al. 2011; Auer and King 2014; Virkkala et al. 2014), and they may reside in the same location long enough to exhibit behavioral adaptations to the local climate (Delgado et al. 2009). Because of the long-term stability in the distributions of resident birds before 1951 and between 1951 and 1960, we can infer that a type of equilibrium between bird distributions and environmental factors occurred. Following climate change, birds adjust their ranges. Thus, particular bird species may have altered their behavior in response to climate warming but not fast enough (Devictor et al. 2012).

### The agreement between observed and predicted distribution changes

The consistency index can provide several pieces of crucial information for the detection and attribution of observed changes in bird distributions. When the changes in distribution result from climate change, these changes should be consistent with the changes predicted solely by climatic factors. When there are many observed changes and the consistency index is low, the observed changes are likely the result of other factors. Conversely, when there are few observed changes and inconsistencies with the predictions, factors other than climate change may have influenced the distribution. However, the consistency index does not fully explain the observed distribution changes because errors or bias in observations and predictions will affect the outcome. A comparison between Figs.2 and 7 shows that the observed distribution changes of some bird species or subspecies appear to be greater than the changes in suitable conditions. Particularly, the species labeled A1 to A5 and I1 to I5 greatly changed from localized to widespread populations, with minor fluctuations in suitable areas. The observed distribution and predicted distribution biases or changes in land use may have caused the changes. The observed and predicted distribution biases of the bird species or subspecies may influence the observed and predicted distribution of the bird species or subspecies (Green et al. 2008). Land use changes are important drivers of biotic change, and they can have positive or negative effects on the availability of resources for bird species; and they can also represent barriers to the dispersal of species (Delgado et al. 2009).

### The attribution of changes in bird distributions

Birds have altered their niches over a century of climate change (Tingley et al. 2009). As expected, our results showed that changes in the northern limits and latitudes of the centers of distribution of nearly half of the subspecies studied can be attributed to climate change over the past 50 years in China. Climate change forces heterogeneous shifts in avian elevation ranges (Tingley et al. 2012). Our results unexpectedly showed that changes in the eastern and western limits and the longitudes of the centers of distribution of very few subspecies of birds can be attributed to climate change. In fact, increasingly more reports indicate other types of range shifts, such as east–west directional shifts across longitudes or, unexpectedly, shifts toward tropical latitudes and lower elevations (Lenoir and Svenning 2015). The different responses may be related to the ecological characteristics of particular bird subspecies or species. In our study, *Aviceda leuphotes leuphotes*, *Accipiter trivirgatus indicus*, *Accipiter badius cenchroides*, *Accipiter badius poliopsis*, *Ictinaetus malayensis*, *Spilornis cheela burmanicus, Spilornis cheela ricketti*, *Spilornis cheela burmanicus, Spizaetus nipalensis nipalensis*, *Chrysolophus pictu*, *Amaurornis akool coccineipes*, *Streptopelia chinensis chinensis,* and *Streptopelia chinensis formosa* distribution changes are attributed to climate change. The bird species or subspecies are usually distributed within forests, grasslands, farmland, and other land in tropical, subtropical or broad warm climate zones; the species are sensitive to climate factors, particularly high-heat propagation conditions that increase the likelihood of migrations (Gao 1996). The characteristics may cause the bird species or subspecies to be more prone to change their range margin than others following climate change. However, *Aviceda leuphotes wolfei, Aviceda leuphotes syama*, *Accipiter trivirgatus formosae, Spilornis cheela hoya, Spilornis cheela rutherfordi, Spizaetus nipalensis orientalis,* and *Streptopelia chinensis hainana* distribution changes are not caused by climate change. These bird species or subspecies are usually distributed in tropical, subtropical or warm, narrow climate zones, and inhabit forests or other land types (Gao 1996). The characteristics may increase the difficulty for these bird species or subspecies to change their range margin than others following climate change. Furthermore, the effects of human activities, sampling bias, the natural spread of species, and that the species distribution change lags behind climate change may influence the results (La Sorte and Jetz 2012). However, the evidence in our study is insufficient.

Different methods have been used to attribute the distributional changes of animals to climate change. For example, Nunes et al. (2007), who used a correlative approach to test a hypothesis of the causation of observed shifts because of a reduction of habitable areas of blue-winged macaws, eliminated climate change as a likely explanation, and revealed the likelihood of other causes. Using a sample-based approach in an elevation gradient in the Italian Alps and data from two recent atlas surveys performed on a 1 × 1 km grid in an 11-year interval, Popy et al. (2010) modeled the elevation gradient of avifaunal compositions and tested whether bird assemblages are shifting upward in elevation synchronously with current climate warming and/or habitat changes. Fine-scale bird-breeding atlases in mountainous regions, along with ordination methods, act as a sensitivity tool to test and measure elevation shifts in species ranges; however, the observed elevation shift in the distributions of the avifauna cannot unambiguously be attributed to climate warming. This shift is smaller than expected based on the regional increase in temperature; thus, how closely bird distributions match climate change is questionable. Maggini et al. (2011) used response-curve shapes to detect elevation shifts and analyze whether birds are tracking climate change in Switzerland. These studies demonstrate that attributing distributional bird shifts to climate change is a challenge. We employed methods that sufficiently attribute changes in bird distributions to climate change, and these methods may also be used to attribute changes in distributions of other animals to climate change.

## Supporting Information

**Appendix S1.** The sources of data and records for the bird distributions.

**Appendix S2**. Climate change in China over the past 50 years.

**Appendix S3.** The membership function form for different climatic factors. **Appendix S4**. Climatic parameters of different subspecies of birds. **Appendix S5**. The membership function parameters of different birds.

**Appendix S6**. The weight coefficient of climatic factors. **Appendix S7**. Model performance by Kappa statistics for different bird species or subspecies. **Appendix S8**. The degree of grey incidence of observed changes at the southern and northern boundaries of distributions of birds with different climatic factors. **Appendix S9**. The degree of grey incidence of observed changes at the eastern and western boundaries of bird distributions with climatic factors. **Appendix S10**. The degree of grey incidence of observed changes in latitude and longitude of distribution centers of the birds with climatic factors.
