# Supplementary material for: Can changes in the distributions of resident birds in China over the past 50 years be attributed to climate change?
Source: Ecol Evol. 2015 May 11;5(11):2215–33. doi: 10.1002/ece3.1513 (PMC4461423; doi:10.1002/ece3.1513)
Supplement: Supplementary file 1 [file ece30005-2215-sd1.doc]

**Appendix S1. The sources of data and records for the bird distributions**

**1 .National level distribution date and records**

Bate L,Cao G,Cao L.(2005).The report on the Yangtze River waterfowl survey. China forestry publish house, Beijing,China.

Caldwell HR,Caldwell JC.(1931).South China birds.Heyster May Vanderburgh,Shanhai,China.

Cheng TH (1947) Checklist of Chinese birds.Trans.Chin.Assoc.Adv.Sci, 9,40-84

Cheng TH (1947).List of recent literature on Chinese ornithology.Biol.Bull,Fukien Chr.Univ.6,107-136

Cheng TH (1976) A Checklist of the Avifauna of China. Science Press, Beijing,China

Cheng TH (1987). A Synopsis of the Avifauna of China. Science Press, Beijing,China

Cheng TH (1994). A complete check list of species and subspecies of the Chinese birds. Science Press, Beijing,China

Cheng TH (2000). A complete checklist of species and subspecies of the Chinese birds. Science Press, Beijing,China

Cheng TH, Tan YK, Lu TC, Tang CZ, Bao G.J, Li FL. (1978).Fauna Sinica.Aves Vol.4 .Galliformes . Science Press, Beijing, China

Cheng TH, Xian YH, Guan GY. (1991).Fauna Sinica.Aves Vol.6 .Columbiformes , Psittaciformes,Cuculiformes and Strigiformes .Science Press, Beijing, China

Cheng TH, Zhang YS, Xian YH. Lu TC, Tan YK, Long ZY, Wang ZY. Tang CZ, Zhou FZ, Liu CJ, Liu RS, Bei TX,Ding WN, Zhang TX,Zhou HZ, .Qian GZ,He JJ,Zhao TA,Li FL (1979) .Fauna Sinica.Aves Vol.2. Anseriformes . Science Press, Beijing, China

Cheng TH,Long ZY.,Zheng BL.(1987).Fauna Sinica.Aves Vol.11.Passeriformes,Muscicapidae Ⅱ .Timallinae. Science Press, Beijing, China

Cheng TH. (1949).On the geographical distribution of bird in China. Peking Nat, Hist, Bull, 18:45-57

Cheng TH. (1950).A study of the geographical distribution of bird in China. The Chinese Journal of Zoology, 4, 83-108

Cheng TH. (1955) the list of birds in china. Science Press, Beijing, China

Cheng th. (1959) China Animal Atlas - 1-3 . Science Press, Beijing, China.

Cheng TH. (1963).Economic birds of China (1nd edn). Sciences Press,Beijing,China.

Cheng TH. (1993).Economic birds of China (2nd edn). Sciences Press,Beijing,China.

Cheng TH. Long ZY, Lu TC (1995).Fauna Sinica.Aves Vol.10 .Passeriformes,Musci capidae Ⅰ .Turdinae. Science Press, Beijing, China.

China Ornithological Society waterfowl group (1994).Waterbird research in China. East China Normal University Press, Shanghai,China.

China Wildlife Conservation Association.(1995).Chinese bird guides.Henan Science and Technology Press ,Zhengzhou,China

China coastal waterbird survey project team.(2011) The report on Chinese coastal waterbird survey (1.2008-12.2009). Hong Kong Bird Watching Society Limited, Hong Kong,China

Fu TS,Song YJ,Gao W,Zheng BL, Wang ZX, Li DH,Wu ZK (1998).Fauna Sinica.Aves .Vol.14 .Passerif ormes : Ploceidae and Fringillidae. Science Press, Beijing, China.

Gao W.(1996).Ecology of Falcon Order in china. Science Press,Beijing,China.

La TouchÉ,JDD.(1925-1934) .A handbook of the birds of eastern China.Taylor And Francis.

Li GY, Zheng BL,Liu G.Z. (1982).Fauna Sinica.Aves Vol.13: Passeriformes (Paridae- Zosteropidae). Science Press, Beijing, China

Li XY,Lian L,Gong P,Liu Y,Lian FF.(2012).Bird watching in china reveals bird distribution chang.Chin Sci Bull,57(31):2956-2963.

MacKinnon J, Phillipps K, He FQ (2000).A Field Guide of the Birds of China. Hunan Education Press, Changsha,China

National Forestry Administration in China (2009).The investigation of the important wild animal resources in china. China forestry publish house, Beijing, China.

Shaw TH (1936) The birds in hopei province.two volumes. Peiking:Fan Memorial Institute of Biology.

Xu WS.(1995)Raptors of china. China forestry publish house, Beijing, China.

Yan CW, Zhao ZJ, Zheng GM, Xu WS, Tan YK (1996).A field guide of birds of China. Kingfisher Culture Co. Ltd. Press, Taipei

Zhang FY,yang RL.(1997).Chinese bird migration study.Chinese forestry publish house,Beijing,China

Zhang RZ (1999).Zoogeography of China. Science Press, Beijing, China

Zhao ZJ (2001). The Avifauna of China. Jilin Scientific Publishing House,Changchun,China

Zheng BL,Yang L,Yang DH . Kuang BY. Wang ZX, Li DH,Wang XT,Zheng GM,Zheng ZX,Li GY,Xu WS,Fu TS.(1985).Fauna Sini ca.Aves Vol.8 . Science Press, Beijing, China

Zheng GM (2005).The classification and distribution list of birds in china. Science Press, Beijing, China.

Zheng GM (2011).The classification and distribution list of birds in china .2^nd^ ed. Science Press, Beijing, China.

Zheng GM,Wang QS (1998). China red data book of endangered animals aves.Science Press ,Beijing, Hong Kong, New York.

**2.Local or regions level distribution date and records**

Agriculture and Forestry Bureau of Tianjin,Tianjin Zoological Society.(1984).Tianjing birds. Tianjin People's Fine Arts Publishing House,Tianjin,China

Anhui Provincial Forestry Department.(2006).Anhui Province terrestrial wildlife resources.Hefei University Press,Hefei,China

Anhui Provincial Revolutionary Committee of Agriculture and Forestry Bureau of Wildlife Resources Investigation Office (1978).Investigation and protection of wildlife resources. Episode.He fei, Anhui Provincial Revolutionary Committee of Agriculture and Forestry Bureau of Wildlife Resources Investigation Office.hefei. ,China

Cai Q K (1987) The Avifauna of Beijing.Beijing publish house,Beijing,China

Chen SH, Huang Q, Fan ZY, Chen CS, Lu.YW (2012)The update of Zhejiang bird checklist. Chinese Birds, 3(2):118–136

Cheng HZ.( 2010)Gaoligongshan Bird Sanctuary and Supplements. Intellectual Property Press, Beijing,China

Cheng SL,Liu JN,Zhang YY.(2011). The birds of Wuyishan national nature reserve. Beijing: Science Press,Beijing,China

Cheng TH, Li DH. Wang ZX. Wang ZY, Jiang ZH, Lu TC (1983).The avifauna of Xizang. Science Press, Beijing,China

Cheng TH, Qian YW, Tan YK. Zheng BL, Guan GX, Li GY, Min ZL,Chen FG,Zhao TR and Shi DQ.(1973).The avifauna of Qinling mountain . Science Press, Beijing. ,China

Cheng TH, Zheng BY XI YH zhou FZ,et al(1961).The investigation of birds in Hunan part two- Passeriformes.Acta Zoological Sinica.13(1-4):97-121.

Cheng TH,Feng ZJ,Zhang RZ,Hu SQ.(1981).On the land-vertebrate fauna of Qinghai-Xizang Plateau with considerations concerning its history of transformation .Memoirs of Beijing Natural History Museum .9 :1- 17

Cheng TH,Qian YW,Zheng BY,XI YH et al(1960).The investigation of birds in Hunan part one-non Passeriformes.acta zoological sinica.12(2):293-319

Cheng TH,Zheng BJ.(1960).The investigation of birds in southern of yunnan province.Acta Zoological Sinica.12(2):250-277

Cheng TH.(1940). The study on the birds in Minjiang River Basin. 1. Journal of biology in xiehe University.1,56-72

Cheng TH.(1944).The report of birds on three years (1937 to 1941) Field observations in Shaowu. Journal of biology in xiehe University,4,63-150

Cheng TH.(1958) The study on the mainly insectivorous fruit birds in Changli. science press,Beijing. ,China

Deng XJ,Wang B,Zhong FS. (2013).Fauna Hunan, Passeriformes, aves in Hunan, China. hunan science and technology publish house,Changsha. ,China

Deng XJ (2007).Monitoring of vertebrate and bird resources in Dongting Lake.Hunan Normal University Press,Changsha,China

Ding P,Fang ZF,Cheng SH. (2012).Birds of the thousand island lake. Higher Education Press, Beijing. ,China

Du TK. (2012)Wetland birds in sand lake of Ningxia. Ocean Press,Beijing,China

Fang LS. (2008).The birds in Shan Xi Province. Chinese forestry publish house,Beijing,China

Fu JW(2007). Ningxia bird guides.Ningxia People's Publishing House,Yinchuan,China

Fu TS.(1984).Birds in Chang bai mountains.Northeast Normal University Press,Changchun,China

Fu ZP (2013) Common bird watching field guide to the Yangtze River Basin.China Environmental Press,Beijing,China

Gang Y,Fang BH.(2004).Investigation and protection of wildlife resources in Henan Province. Yellow River Conservancy Press, Zhengzhou,China

Gao W.(2006). Studies on birds and their ecology in Northeast China. Science Press,Beijing,China

Gao ZX.(1989).Zhalong birds.Chinese forestry publish house. Beijing,China

Gu W,Zhu CY,Liu JS,Fu BZ,Li GZ.(1980).The investigation of birds in yangzhou.Journal of jiangsu agricultural university,1(3):61-68

Guangdong Institute of insects animal room, Zhongshan University,Department of Biology (1983) Hainan Island birds and beast.Science Press,Beijing,China

Guizhou fauna Editorial (1980). Guizhou vertebrate distribution list.Guizhou People's Publishing House,Guiyang,China

Heilongjiang Provincial Institute of Wildlife.(1992) The avifauna of Heilongjiang. China Forestry Publishing House,Beijing,China

Henan Sanmenxia Yellow River Wetland National Nature Reserve Management Office. (2012).Yellow River wetland birds – Sanmenxia.Henan Science and Technology Press, Zhengzhou,China

Hu HX,Tang RC,Tang RY,Cheng Y.(1978).Birds In Guizhou. Journal Of Wuhan University,2：67-77

Hu HX,Wang H.(1995).Diversity and Protection of Hubei birds.Wuhan University Press, Wuhan,China

Huang MP.(1989).Liaoning fauna-birds.Liaoning Science and Technology Press. Shenyang,China

Huang ZY, Song Z.H, Yu K (1993).The birds in Shanghai. Fudang University publish house, Shanhai,China.

Jigongshan National Nature Reserve Administration, Xinyang City, Henan Province, the Wildlife Conservation Society.(2013) The birds of Jigongshan.China Forestry Publishing House,Beijing,China

Jilin Wildlife Conservation Society (1987) Jilin Wildlife Atlas.Jilin Science and Technology Press,Changchun,China

Joint Committee on Northeast Wildlife protection.(1988).Birds in Northeast.Liaoning Science and Technology Press, Shenyang. ,China

Kunming Institute of Zoology,Chinese Academy of birds Group(1980).Gaoligongshan region vertebrate inspection report. Volume 2- birds. Science Press. Beijing. ,China

Lei JY,Zhang SY,Zhang SY,Zhang XM.( 2012).Latest Number of Bird Species of Hubei Province. Sichuan Journal of Zoology ,31( 6):987-991

Li GH (1995) The birds in Si Chuan province.Chinese forestry publish house, Beijing,China

Li GH (1995)The colour handbook of the birds of Sichuan.Chinese forestry publish house,Beijing,China

Li GH.(1985)Sichuan resources fauna. (Volume III - Bird).Sichuan Science and Technology Press,Chengdu,China

Li JZ (2012).Dongting Lake Bird Atlas.: Hunan Science and Technology Press,Changsha,China

Li QW,Zhang FJ.(2007)An illustrated guide to the bird of northeast China. Liaoning Normal University Press,Dalian,China

Liaoning Bird Investigation Team.(1986) Liaoning Bird Survey Report.Liaoning University Press,Shenyang,China

Liaoning Province Forestry Society (1984).Liaoning major economic Birds Atlas.Liaoning Science and Technology Press,Shenyang,China

Liaoning Provincial Institute of Science of Sericulture.(1995).Liaoning silkworm breeding bird.Liaoning Science and Technology Press,Shenyang,China

Liu NF,Bao XK,Liao JC.(2013)The classification and distribution of the birds in Qingzang plateau. science press, Beijing,China.

Liu XY, Ding ZX, Wang HW. (2010).Aves in Taiwan (1-3) .Taiwan's Council of Agriculture, Forest Service,Taipei.

Liu XZ (2011).Beidaihe birds Illustrated Record.Hebei Education Press,Shijiazhuang,China

Liu YL (2013) .Birds of the Yellow River Delta.China Forestry Publishing House,Beijing. ,China

Luo JS,Xu J (2013).Lushan common birds. Volume Ⅰ.Jiangxi Science and Technology Press ,Nanchang,China

Ma M (2011) .A checklist on the distribution of the birds in Xingjian.science press,Beijing,China

Msar Had Landscaping Department, South China Institute of Endangered Animals (2010)Birds of Macao.Macau IACM Landscaping Department,Macao. ,China

Nie YQ.(2011) Wild birds in Inner Mongolia. Encyclopedia of China Publishing House,Beijing. ,China

Niu HX,Lü JQ,Lu JQ,Bu YZ,Zhang XF,Zhang KY,Zhu JG.(2002).Survey on the Carnivorous Birds in Henan Province.Chinese Journal of Zoology, 37(1):36-38

Northwest Plateau Institute of Biology, Chinese Academy of Sciences (1989). Economic Fauna in Qinghai Qinghai People's Publishing House,xining,China

Peng KF,Shao YG.(2013)Lushan Birds Collection.China Drama Press,Beijing,China

Peng Y Z (1983) A checklist of yunnnan province birds.Yunnan publish house, Kunming. ,China

Peng YZ (1987)Yunnan birds List. Yunnan Science and Technology Press. Kunming. ,China

Qi YP,Cheng XL.(2012) Daqing Longfeng wetland Bird resource survey and rational development and utilization.Northeast Forestry University Press,Harbin,China

Qian YW,Zhang J,Zheng BJ,Wang S, Guang GX.& Shen XZ.(1965) The mammals and birds investigate in southern of Xin jian province, china. Science press, Beijing, China.

Sai DJ,Sun YG. (2013).A checklist and distribution of the birds in Shandong.science press,Beijing.

Shao MQ,Dai NH,Zhao S,Guo QX,Zhong PH. (2010).latest number of birds in jiangxi province.si chuan journal of zoology,29(3):459-460

Shi ZR.(1992).Huang hai bird.Jiangxi Fine Arts Publishing House,Nanchang,China

Sichuan Resources Fauna Editorial Board. (1985).Sichuan resources fauna. Vol 3.birds.: Sichuan Science and Technology Press,Chengdu,China

Song JC. 2009.Shenzhen wild birds.Sichuan University Press,Chengdu,China

South China Institute of Endangered Animals.(1991).Guangdong birds color illustrations.: Guangdong Science and Technology Press,Guangzhou.,China

Sun CQ,Wang WY,Xu ZW,Feng N,Zhang L (2007).Primary Report On Avian Investigation In Shaanxi Province. Acta Zootaxonomica Sinica 32 ( 4) : 993- 995

Sun CS. (2007)The illustrated encyclopedia of birds of Shaanxi, China.Shaanxi Science and Technology Press,Xi'an,China

Tang CZ (1996).Birds of the Hengduan Mountains Region . sciences Press, Beijing,China

Tang ZH,cheng YL,Tang RG.(1996).A study of avifauna of fujian province.Journal of Fujian Normal University12(2):77-87

Wang FQ.(2006) Annals of Tianjin-the Birds. Tianjin Academy of Social Sciences Publishing House,Tianjin. ,China

Wang J X, Wu S X, Huang G Y, Yang X Y,Cai Z H ,Cai M Q and Xiao,QL (1991).A Field Guide of Birds of Taiwan. Taiwan Wild Bird Informat ion Press,Taipei.

Wang TH,Qiang GZ.(1988) Yangtze Delta Hangzhou Bay shorebird. East China Normal University Press,Shanghai.,China

Wang XP.( 2010)Avian ecology in wetland of Hangzhou bay. China Academy of Fine Arts Publishing House,Hangzhou,China

Wang XT (1990) The Fauna of Vertebrates in Ningxia Province. Gansu Science and Technology Press, Lanzhou,China

Wang XT (1991) The Fauna of Vertebrates in Gansu Province.: Gansu Science and Technology Press, Lanzhou,China

Wang ZQ. (2010).Yulin birds.Shaanxi Science and Technology Press,Xi'an,China

Wu FS.(2004).Qinghuangdao birds.Chinese forestry publish house,Beijing,China

Wu MC. (1993).Guangxi wildlife.Guangxi People's Publishing House. Nanning,China

Wu ZK,Cheng Y,Wu SE,Lin QW.(1979).The birds in guizhou.Journal of Wuhan University 4;52-112

Wu ZK,Lin QW,Yang JL,Liu JC,Wu L(1986).The Avifauna of Guizhou Province.Guizhou Peoples Publishing House, Guiyang,China

Xie DL.(2012)Kashi wild bird guides.China National Photographic Art Publishing House,Beijing,China.

Xing LL (1996).Wuliangsu Lake of the Birds.Inner Mongolia University Press,Hohhot,China

Xinjiang Academy of Sciences expedition comprehensive animal group.(1959).Preliminary investigation report, Xinjiang Tianshan Mountains mammals, birds, and major livestock parasitic worms. science press. Beijing,China

Xu SG.(1991).birds in si chuan.Sichuan Nationalities Publishing House,Chengdu. ,China

Xu WS, Williams M. (1990) Beijing Museum of Natural History research reports. NO 47-Beidaihe Bird Observatory and Research. Beijing Science and Technology Press. Beijing,China

Xun XM.(2012) Wild birds in Shenyang area. Northeast Forestry University Press,Harbin. ,China

Yan CW (1996) A Field Guide to the Birds of China.Kingfisher Culture Co .Ltd .Press, Taibei 1-521.

Yang GS,Xing LL.(1988).Inner vertebrate distribution list .Inner Mongolia University Press.huhehaote,China

Yang L (1995).The Avifauna of Yunnan China.Vol.Ⅰ.Non-Passerif ormes . Yunnan S science and Technology Press, Kunming,China

Yang L,Yang XJ.(2004) Avifauna in Yunnan, volume II.passerine.Yunnan Science and Technology Press,Kunming,China

Yang ZJ, Li JS. (1996).The resources of birds investigation in west Hunan province. Journal of Jishou university 16(5):61-66.

Yang ZJ,Li JS.(1994).The resources of birds investigation in west hunan province.Journal of jishou university 15(6):90-92.

Yu XP,Li JG.(2012) A field guide to the birds of Qinling Mountains.,science press,Beijing,China

Yuan GY (1989) Xinjiang vertebrate Fauna.Xinjiang People's Publishing House,wulumq,China

Yuan XF (2011).Dongzhai bird guides.Henan Art Publishing House,Zhengzhou,China

Yue HQ (1988).Wildlife.-birds and brester. In Yichun .Jiangxi College Press,Nanchang,China

ZhangJF.(1997). Sichuan Bird Identification Guide.Chinese forestry publish house,Beijing,China

Zhang WF (1990).A Field Guide to the Birds of Taiwan. Bird Films & Books Co .Ltd .Press, Taibei.

Zhao ZJ. (1985).Changbai the Birds.Jilin Science and Technology Press,Changchun,China

Zheng WS. (1994).Northwest China rare and endangered fauna. China Forestry Publishing House, Beijing,China

Zhou F (2011).Guangxi terrestrial vertebrates distribution list .:Chinese forestry publish house, Beijing,China

Zhou F.(2010) Chinese mangrove birds.,science press, Beijing,China

Zhou YH, Zhou B, Lin XL. (2009) A checklist on the distribution of the birds in xinjiang.xin jiang people publish house,Wulumq,China

Zhu X,Jiang HL,Lv YC.(2008).A complete taxonomic checklist and geographic reference of bird species and subspecies in Eastern China.science press,Beijing,China

Zhuge Y (1990).Fauna of Zhejiang-Aves. Zhejiang Science and Technology Press, Hangzhou,China.

Zoological Society of Guangxi Zhuang Autonomous Region.(1988).Guangxi terrestrial vertebrates distribution list. Guangxi Normal University Press,Nangning,China

Ge JW,Cai QH,Hu HX,Liu JK,Cao GB,Zhu ZQ,Liu SX,Shi DL (2004)The studies of water fowls resource in hubei province.journal of natural resource 19（3）:285-292.

**3.Natural reserves level distribution date and records**

Bo SQ, Tang SX,Wang JF,Yue T,Chen Y (2010).Bird Biodiversity of Tiantong National Forest Park, Zhejiang Province. Chinese Journal of Zoology 45(5) :86 -94.

Chen LM,Ouyang WF.(2001).Birds in National Nature Reserve of Tangjiahe,Sichuan.Chinese Journal of Zoology 36(4):63-66

Chen ZQ,FU JP,Zhao XR,Ding CQ.( 2010)The Construction of Birds in Yuanmingyuan Relic Park,Beijing. Chinese Journal of Zoology ,45(4) :21-30

Cheng SL,LIN JS.(2011)A Survey on Avian Diversity in Wuyishan National Nature Reserve,Jiangxi. Chinese Journal of Zoology 46( 5) : 66-73

Ding YH. Liu B.(2004)Dafeng Milu National Nature Reserve of Wild Fauna and Flora List. Nanjing Normal University Press,Nanjing,China.

Hu J,hang LX..(2007).Study on Bird Fauna of Tongbiguan Nature Reserve. Forest　Inventory　and　Planning, 32(2):54-57

LI HJ(2003).The study of biodiversity in da mingshan natural reserves. Journal of Xinyang Normal University 16(1) :62-67

Liu XS,Fang Y, Shi XJ.(2003) Present situation and protection counter measures of bird resources at Lianhua mountain nature reserve in Gansu province. Journal of Gansu Forestry Science and Technolog,28(4):27-29.

Liu ZS,Teng LW,Song YL,Li SY,Fu YN,Zeng ZG (2004).A Preliminary Research of Avifauna of Hainan Datian National Nature Reserve, Hainan, China.Chinese Journal of Zoology, 39( 1) : 93-99

Ran JC,Chen HM,Xiong ZB. (2003)A Research of Avifauna in the Maolan Nature Reserve.Gui zhou Forestry Science and Technology,31（3）：26-33

Shao MQ,Zeng BB,Xu Xz,Shang Xl,Dai NH.(2013).Preliminary study on bird composition and diversity in Poyang Lake watershed .during non-breeding period.,33(1):140-149

Wang K.(2003).China national level natural reserve. An Hui science and technology press.hefei,china.

Wu YH,Wu YF.(2005)A Preliminary Study of Birds in Xingtai Forest Park.Sichuan Journal of Zoology,24(4),562-567

Xu LJ, Wang Y ,Chen XL.( 2002)Ecological Analysis of Summer Avian Community in the National Natural Reserve of Meihua Mountain. Journal of Xiamen University (Natural Science), 41(3):364-369.

Xu Y,Ran JH,Yue BS.(2008) The Updated Number of Species of Birds in Sichuan. Sichuan Journal of Zoology ,27(3) ,429-431

You B,Yu X,G,Yang L,Wang X H.(2003).The resources of raptors and protection in deyang country. Journal of Sichuan Forestry Science and T echnology,24(2):53-56

Zhang J,Yang ZS,Huang XF,Zhou CQ.(2009) Initial investigation on the birds in dongyang nature reserve,sichuan.Journal of China West Normal University, 30(4):327-353

Zhou JG,Zheng ZX,Niu XN,Peng YS,Xiao HM,Yang YP,Deng JM,Hong HZ (2013)Survey of Bird Resources in Huizhou.Journal of Anhui Agri.Sci,41( 14) : 6285-6287

**4.New records of distribution date**

Cai YT,Tang SM,Yuan X,Wang JY,Ma ZJ.(2011)Checklist and Change of Birds in Shanghai。Journal of Fudan University 50（3）:334-343.

Chen QH,Zhang R,Huang B J,Yu W J,Cai FK,Feng X C,Li BY,Piao ZJ.( 2014)The new records of birds from Changbai mountain national nature reserve of Jilin. Journal of Jilin Forestry Science And Technology 43(6):28-29

Ci B,Lu WB,Yuan DJ,Li H,Yang Y,Wang XS.(2007).The new records of Brown Crake in dongzhai natural reserve.Chinese Journal of Wildlife 28（3）:71

Dai NH,Liu W,Cai WL,Lin Q (1996).The New records of birds in jiangxi province.Chinese Journal of Zoology,31( 2) : 48-49

Du Y,Zhou F,Shu XL *et al* .(2009) The impact of global warming on china avifauna.Acta Zootaxonomica Sinica, 34,664-674.

Fei YL, Lei M, Zhang Y, Lu CH. (2010).Geographic Distribution Change of Crested Goshawk. Chinese Journal of Zoology, 45(3):174 - 175

Gao XB,Zhao HF,Luo SY,Luo L,Hou YB. (2008)Avifauna Changes in 30 Years ( 1977 to 2007) in Xian, China. Chinese Journal of Zoology,43( 6) : 32- 42

Gong HS,Ma YS,Zeng ZG,Cai XL,Chen S H (2007) Investigation on Resource of Birds in Qinling and DabashanMountains, Shaanxi. Sichuan Journal of Zoology ,126(14),746-759

Gu HS,Gao XB,Li JQ.( 2007)The two new records of birds in shangxi province. Chinese Journal of Zoology, 42(4) : 146.

Gu HS,Zeng ZG,Wang XF,Zhao KH,Zhang Q. (2009) State Key Status and Analysis of Shaanxi Province to protect wildlife.journal shang xi normal university,37(1):52-59

He FQ,Lin JS,Wang YY,Wang GF,Hong YH, Zheng P Ji,Wen C,Lin Z,Shi QH (2014).Bird records from Wuyuan,NE Jiangxi of SE China. Chinese Journal of Zoology,49( 2):170-184.

[Hu](http://epub.cnki.net/kns/popup/knetsearchNew.aspx?sdb=CJFQ&sfield=作者&skey=胡小龙&scode=) Xl,[Geng](http://epub.cnki.net/kns/popup/knetsearchNew.aspx?sdb=CJFQ&sfield=作者&skey=耿德民&scode=) DM. (1995)The new records of birds in an hui province- Black Baza. Chinese Journal of Zoology,30(5):24-25

Huang J ,Fu JG, Guo YM (2011)The new records of birds in hei long jiang province-Crested Serpent Eagle .Sichuan Journal of Zoology,30(6):881

Jiang MD,Wu XZ (1988)New records of Hodgson’s Hawk Eagle in sichuan Province.Sichuan Journal of Zoology, 7(2):14

Lei JY,Zhang Ly,Zhang SY,Zhu XM (2012)Latest Number of Bird Species of Hubei Province.Sichuan Journal of Zoology ,31(6):987-991

Li LX,Zhang HJ.(2006).Present Situation of Study on Chrysolophus pictus.Sichuan Journal of Zoology,25(14): 2006

Li XZ,LI R, Yuan ZH, Shen F (2011).New Record of Birds Distribution in Changqing National Nature Reserve. Shaanxi Forest Science and Technolog ( 1) : 41-42

Li ZW (2003)Preliminary Report on Brown Crakes Distribution in Hubei Province.Forest Resources Management,3,44-53

Liao Ck, Lin BZ, Zhang CY ( 2011).New Birds Records of Jiulianshan National Nature Reserve, Jiangxi Province.jiang xi forestry science and technology,2：44-45

Liu G,Li Xg ,Liu XZ (2011).Cataloging the Terrestrial Vertebrate Species in Jiangxi Province. jiang xi forestry science and technology,2,48-61

Liu Y,Wei Q,Dong L,Lei JY.( 2013)On An Update of recent New Bird records in China. Chinese Journal of Zoology,48( 5) : 750-758

Lu CH,Lei M,Zhang Y. (2010).The new records of birds in Jiangsu province- Black Baza、White-breasted Kingfisher. Chinese Journal of Zoology 35(56);89

Ma Q,Li JQ,Zhang ZW. (2008).The new records of birds in henna- Crested Goshawk.Journal beijing normal university 44(6):613-614

Peng B Y,Shu F,Liu S l,Cao F,Deng XJ,Wang B (2014)Fourteen new records of birds in the West Dongting Lake Nature reserve. Hunan Forestry Science ＆ Technology,41(2):12-16

Qian YW .(1963).New records of birds from xinjiang.Acta Zoological Sinica,15(56):168

Shan K,Yu Jb (2013).New Birds record Discovered from Yellow river Delta,Shandong Province. Sichuan Journal of Zoology ,32(4),609-612

Shi CF,Yang GS (2006).A New Bird Record in Hohhot: Spotted Dove Streptopelia chinensis.Zoological Research, 27 ( 6) : 656

Song ZW,Lewthwaite RW, Yu RD, Li GC,Leven MR, Williams MD,Sherred K, Lazell J,lu WH.(2008). China avian fauna on islands and coastal wetlands at the tropic of cancer in june. Acta Zootaxonomica Sinica, 33 ( 1) : 217- 222

Sun QH,Zhang ZW (2000).The Impact of Climate Warming on the Distribution of Chinese Birds.Chinese Journal of Zoology,35,45-48.

Wang J.(2010).Five New Bird Records from Anhui Province, China. Journal of Huangshan University,12(3):52-53

Weng C,Hang D,Song C.(2011) the new records of two raptors in Beijing.Chinese Journal of Zoology,47:142

Weng C,Song H,Heng D,Song X,Ye H (2013).The records of Crested Goshawk and hei chi yuaninbei jing. Chinese Journal of Zoology 6：851

Xiao HF,Deng XZ, Li JD, Liu E, Tian JM, Chen XQ, Yang QR, Dai ZX.(1989).The birds of wudang mountain.The Journal central china normal university 23(1):73-81.

Zhang H,He LW,Yuan M (2006)The records of Hodgson’s Hawk Eagle in wen country of Gansu province.Chinese Journal of Zoology,42,63.

Zhang QJ,Cheng YL,Tang ZH.( 2001).The new records of birds subspecies in Fujiang province.- Crested Goshawk. Sichuan Journal of Zoology 20(2) : 81.

Zhu J,li CH. (2006).The New Records In Shangxi Province-Golden Pheasant.Chinese Journal of Zoology,42,28

**5 .Others resources of distribution date and records**

China Zoological Society.China Bird Report (2003-2010). Beijing: China zoological society of birds

Chinese bird species network database for information retrieval（[http://www.birders.cn](http://www.birders.cn/)）

China Animal Themes Database（http://www.zoology.csdb.cn）.
