# Supplementary material for: Can changes in the distributions of resident birds in China over the past 50 years be attributed to climate change?
Source: Ecol Evol. 2015 May 11;5(11):2215–33. doi: 10.1002/ece3.1513 (PMC4461423; doi:10.1002/ece3.1513)
Supplement: Supplementary file 2 [file ece30005-2215-sd2.doc]

**Appendix S2. Climate change in China over the past 50 years**

To analyze the correlation between change in the distribution of birds and climatic factors, we analyze the climate change trends in China over the past 50 years. The climate change trends from 1961 to 2013 in China have been reported (China climate change bulletin, Chinese Administration of Meteorology 2014). However, some climate factors related to change in distribution of birds have not been analyzed.

**Table S1** The climatic factors

| **No** | **Climatic factors** | **unit** |
| --- | --- | --- |
| 1 | mean annual air temperature | ℃ |
| 2 | mean air temperature in January | ℃ |
| 3 | mean air temperature in July | ℃ |
| 4 | maximum temperature in warmest month | ℃ |
| 5 | minimum temperature in coldest month | ℃ |
| 6 | sums of cumulative temperature above 0℃ | ℃ |
| 7 | the annual mean bio-temperature | ℃ |
| 8 | annual precipitation | mm |
| 9 | annual potential evapotranspiration rate |  |

Because bird distributions are likely to have direct or indirect relationships with the macroclimate and microclimate, the mean annual temperature, mean temperatures in January and July, sum of the cumulative temperatures above 0°C, minimum temperature in the coldest month and maximum temperature in the warmest month were calculated. In addition, annual precipitation and the Holdridge index (Holdridge 1967; Zhange 1993), including the mean annual bio-temperature (BT) and the annual potential evapotranspiration rate (PER), were selected (See Table S1).

Climatic data for the last 50 years in China were provided by the climate center of the Chinese Administration of Meteorology as 17,625 grid cells with a resolution of 0.5°×0.5°. These data included daily air temperature, maximum and minimum temperatures, and daily precipitation. The climate indices(Table S1) in every grid cells were calculated from 1961 to 2010 using Visual Fortran (Compaq Visual Fortran Professional Edition 6.5, Compaq Computer Corporation (2000), Harris County, Texas, US).

**Fig S1. Climate change trend over the past 50 years in China**

Note: a, b, c, d, e, f, g, h, and i represent the mean annual air temperature, mean air temperature in January, mean air temperature in July, the highest temperature in the warmest month, the lowest temperature in the coldest month, sums of cumulative temperature above 0°C, annual precipitation, BT and PER, respectively.

To analyze the relationship between climate change and the changes in bird distributions, the climate data over the last 50 years were analyzed for each decade from 1961 to 2010. We used ordinary kriging methods to convert the climate variables into records of bird distributions based on a digital elevation model (DEM) and produced gridded data for China that were used to overlay the distribution of birds and climate factors in ARCGIS (Vers. 9.2 for Windows, Esri Corp. (2008), Redlands, CA, US). Climatic variables were generated for each distribution point for each bird in each decade.

Over the last 50 years, the mean air temperature in China has generally increased from 5.62°C in the 1960s to 6.89°C in the 2000s. The air temperature in January showed a similar trend, increasing by approximately 1.6°C. Similarly, the air temperature in July increased by approximately 0.82°C. The annual minimum air temperature increased by 1.73°C, and the annual maximum air temperature increased by 1.05°C. In addition, the cumulative temperatures above 0°C increased from 3289.4°C in the 1960s to 3533.9°C in the 2000s. The annual precipitation in China has become increasingly variable, showing a decrease from 603.41 mm in the 1960s to 597.64 mm in the 1970s, an increase to 627.52 mm in the 1990s, and then a decrease to 605.09 mm in the 2000s. The BT tracked the cumulative temperature above 0°C from 8.96°C in the 1960s to 9.62°C in the 2000s. The PER declined from 2.36 in the 1960s to 1.87 in the 1980s and then increased to 2.06 in the 2000s (Fig. S1).

Our results demonstrate that regional climate change in China displays a trend that is similar to the changes in the global climate. Over the period from 1880 to 2012, the global average surface temperatures warmed by 0.85°C [0.65 to 1.06°C] (IPCC 2013). Our results show that over the past 50 years, the annual precipitation in China fluctuated considerably (Fig. S1). The precipitation averaged over the mid-latitude land areas of the Northern Hemisphere has increased since 1901, whereas at other latitudes, there is low confidence in the area-averaged long-term positive or negative trends (IPCC 2013). Our results also indicate that increases in the extreme low and high temperatures are consistent with global warming (IPCC 2013).

**References**

Holdridge,L.R .1967. Life Zone ecology.rev.ed.tropical science center and Jose, Costa Rica.

IPCC.2013.Summary for Policymakers. In: *Climate Change 2013: The Physical Science Basis. Contribution of Working Group I to the Fifth Assessment Report of the Intergovernmental Panel on Climate Change* [eds Stocker,T.F., D.Qin,G-K.Plattner,M.Tignor,S.K.Allen,J. Boschung,A.Nauels,Y.Xia,V.Bex, and P.M.Midgley]. Cambridge University Press, Cambridge, United Kingdom and New York, NY, USA.

Zhang,X.S.1993.A vegetation-climate classification system for global change studies in China. *Quarter.Sci.*2:157-169.
