# Supplementary material for: Can changes in the distributions of resident birds in China over the past 50 years be attributed to climate change?
Source: Ecol Evol. 2015 May 11;5(11):2215–33. doi: 10.1002/ece3.1513 (PMC4461423; doi:10.1002/ece3.1513)
Supplement: Supplementary file 3 [file ece30005-2215-sd3.doc]

**Appendix S3.**The membership functions for different climatic factors

| **Climate**  **factors** | **type** | **The membership function** | **parameter** | |
| --- | --- | --- | --- | --- |
| Air temperature | symmetric | $A\left(x\right)=\frac{1}{1+\alpha\left(x-a\right)^{\beta}}$ （$\alpha>0,\beta$ is positive even number）,x is the mean air temperature, a is the mean of the mean air temperature | α | β |
| Air  temperature  in January | symmetric | $A\left(x\right)=\frac{1}{1+\alpha\left(x-a\right)^{\beta}}$ （$\alpha>0,\beta$ is positive even number）,x is the mean air temperature in January ,a is the mean of the air temperature in January | α | β |
| Air temperature in July | symmetric | $A\left(x\right)=\frac{1}{1+\alpha\left(x-a\right)^{\beta}}$ （$\alpha>0,\beta$ is positive even number）,x is the mean air temperature in July, a is the mean air temperature in July | α | β |
| The highest temperature in the warmest month | monotonically decreasing | $A\left(x\right)=\left\{\begin{matrix} 1 \\ \frac{1}{1+\alpha\left(x-a\right)^{\beta}} \end{matrix}\right.\begin{matrix} x\leq a \\ x>a \end{matrix}$ （$\alpha>0,\beta>0$）,x is the highest air temperature, a is the mean of the highest air temperature | α | β |
| The lowest temperature in the coldest month | monotonically increasing | $A\left(x\right)=\left\{\begin{matrix} 1 \\ \frac{1}{1+\alpha\left(x-a\right)^{-\beta}} \end{matrix}\right.\begin{matrix} x\leq a \\ x>a \end{matrix}$ （$\alpha>0,\beta>0$）,x is the lowest air temperature, a is the mean of the lowest air temperature | α | β |
| Sums of cumulative temperature above 0℃ | symmetric | $A\left(x\right)=\frac{1}{1+\alpha\left(x-a\right)^{\beta}}$ （$\alpha>0,\beta$ is positive even number）,x is the mean of the cumulate temperature, a is the mean of the cumulate air temperature above 0℃ | α | β |
| Annual precipitation | symmetric | $A\left(x\right)=\frac{1}{1+\alpha\left(x-a\right)^{\beta}}$ （$\alpha>0,\beta$ is positive even number）,x is the mean precipitation, a is the mean of the annual precipitation. | α | β |
| BT | symmetric | $A\left(x\right)=\frac{1}{1+\alpha\left(x-a\right)^{\beta}}$ （$\alpha>0,\beta$ is positive even number）,x is the mean of BT, a is the mean of BT. | α | β |
| PER | symmetric | $A\left(x\right)=\frac{1}{1+\alpha\left(x-a\right)^{\beta}}$ （$\alpha>0,\beta$ is positive even number）,x is the mean of the PER, a is the mean of the PER. | α | β |

Note: $A\left(x\right)$ is the membership of the membership function

**Appendix 4 Climatic parameters of different subspecies of birds**

|  | A | B | C | D | E | F | G | H | I | J | K | L | M | N | O | P | Q | R | S |
| --- | --- | --- | --- | --- | --- | --- | --- | --- | --- | --- | --- | --- | --- | --- | --- | --- | --- | --- | --- |
| TP | 17.98 | 11.44 | 25.15 | 18.32 | 20.14 | 22.25 | 7.89 | 19.41 | 17.43 | 15.36 | 24.51 | 20.55 | 15.03 | 3.05 | 14.74 | 18.1 | 14.52 | 24.3 | 17.43 |
| TX | 13.73 | 3.41 | 25.15 | 3.41 | 19.6 | 15.12 | 3.23 | 11.92 | 8.21 | 9.16 | 23.5 | 19.6 | 2.64 | -1.79 | -0.85 | 8.97 | 3.41 | 23.08 | 5.44 |
| TD | 23.68 | 17.79 | 25.15 | 25.04 | 20.82 | 25.3 | 11.53 | 25.8 | 22.28 | 21.88 | 25.3 | 21.77 | 25.15 | 7.5 | 21.12 | 21.52 | 22.59 | 25.8 | 23.68 |
| PY | 7.78 | 1.67 | 19.75 | 9.96 | 11.87 | 15.66 | -10.42 | 10.85 | 7.35 | 8.09 | 19.22 | 12.47 | 5.82 | -18.52 | 3.54 | 7.71 | 3.03 | 18.62 | 10.38 |
| XY | 2.05 | -5.41 | 19.75 | -5.41 | 11.12 | 4.84 | -18.47 | 3.44 | -4.48 | -0.04 | 17.6 | 11.12 | -6.11 | -25.03 | -12.54 | -1.63 | -12.49 | 17.29 | -4.59 |
| DY | 16.88 | 7.33 | 19.75 | 19.58 | 12.82 | 20.83 | -5.47 | 21.59 | 16.43 | 16.01 | 20.83 | 14.38 | 19.75 | -10.72 | 13.33 | 13.89 | 14.92 | 21.59 | 16.88 |
| PQ | 27.11 | 21.31 | 28.42 | 25.27 | 26.64 | 27.27 | 22.64 | 26.47 | 26.5 | 19.48 | 28.49 | 26.8 | 23.49 | 21.53 | 24.52 | 27.66 | 24.75 | 28.47 | 22.13 |
| XQ | 23.11 | 13.88 | 28.42 | 13.88 | 26.36 | 20.83 | 22.23 | 16.17 | 23.62 | 13.53 | 28.4 | 26.6 | 12.25 | 19.4 | 10.07 | 26.43 | 17.72 | 28.35 | 13.88 |
| DQ | 28.73 | 25.89 | 28.42 | 28.91 | 26.8 | 28.91 | 23.81 | 28.91 | 28.58 | 23.89 | 28.91 | 27.23 | 28.58 | 23.54 | 28.75 | 28.73 | 28.75 | 28.91 | 26 |
| GP | 38.41 | 34.74 | 37.75 | 37.03 | 36.08 | 37.51 | 38.74 | 37.36 | 38.07 | 33.38 | 37.56 | 36.24 | 35.65 | 37.43 | 37.76 | 38.52 | 37.69 | 37.64 | 35.45 |
| GX | 35.52 | 30.1 | 37.75 | 30.1 | 35.61 | 33.56 | 38.08 | 30.8 | 36.07 | 27.83 | 37.32 | 35.76 | 26.28 | 35.95 | 25.61 | 37.47 | 32.99 | 37.03 | 30.1 |
| GD | 40.49 | 38.97 | 37.75 | 40.66 | 36.28 | 38.9 | 39.92 | 39.71 | 39.41 | 37.42 | 37.79 | 36.72 | 39.71 | 40.87 | 41.28 | 39.72 | 40.95 | 38.17 | 40.33 |
| XP | -6.63 | -10.82 | 5.24 | -4.2 | 3.61 | 1.21 | -30.71 | -2.63 | -7.28 | -5.39 | 4.44 | 4.11 | -9.08 | -37.36 | -10.44 | -6.41 | -11.44 | 4.35 | -3.76 |
| XX | -17.27 | -26.59 | 5.24 | -26.59 | 2.75 | -8.13 | -44.78 | -14.7 | -22.53 | -15.64 | 1.94 | 2.5 | -25.47 | -45.45 | -33.9 | -21.86 | -31.95 | 1.39 | -26.59 |
| XD | 2.2 | -2.06 | 5.24 | 5.8 | 4.54 | 6.1 | -23.8 | 5.8 | 1.73 | 1.26 | 6.1 | 5.73 | 5.24 | -28.85 | 0.03 | 1.01 | 2.19 | 5.8 | 1.73 |
| JP | 6592 | 4478 | 9078 | 6742 | 7115 | 8148 | 3769 | 7029 | 6441 | 5410 | 8983 | 7276 | 5547 | 2945 | 5388 | 6636 | 5377 | 8895 | 6369 |
| JX | 5366 | 2356 | 9078 | 2356 | 6950 | 5698 | 3228 | 3946 | 4170 | 2960 | 8690 | 6950 | 1801 | 2335 | 1198 | 4474 | 2961 | 8620 | 2356 |
| JD | 8228 | 6260 | 9078 | 9062 | 7332 | 9252 | 4130 | 9392 | 8058 | 7759 | 9252 | 7754 | 9078 | 3511 | 7727 | 7827 | 8211 | 9392 | 8010 |
| SP | 1530 | 1299 | 1162 | 1425 | 962 | 1589 | 142 | 1435 | 1487 | 1028 | 1647 | 1009 | 1323 | 545 | 1073 | 1580 | 1095 | 1678 | 1214 |
| SX | 801 | 659 | 1162 | 659 | 932 | 962 | 69 | 823 | 588 | 435 | 962 | 936 | 435 | 292 | 362 | 1272 | 189 | 962 | 574 |
| SD | 2002 | 1742 | 1162 | 2431 | 985 | 2272 | 233 | 2057 | 2382 | 1523 | 2071 | 1233 | 2326 | 863 | 1958 | 2326 | 2326 | 2272 | 1661 |
| WP | 18.02 | 12.91 | 23.83 | 18.44 | 18.29 | 21.97 | 10.11 | 18.84 | 17.58 | 14.01 | 24.14 | 18.83 | 15.16 | 8.05 | 14.61 | 18.17 | 14.68 | 24.02 | 17.24 |
| WX | 14.38 | 6.75 | 23.83 | 6.75 | 17.69 | 15.76 | 8.9 | 9.18 | 11.19 | 6.49 | 23.83 | 17.94 | 5.5 | 6.42 | 3.48 | 14.92 | 7.58 | 23.39 | 6.75 |
| WD | 22.38 | 16.32 | 23.83 | 24.52 | 18.89 | 24.54 | 10.54 | 24.99 | 21.86 | 20 | 24.54 | 20.15 | 23.88 | 9.59 | 21.33 | 21.09 | 21.94 | 24.99 | 19.77 |
| ZP | 0.73 | 0.58 | 1.36 | 0.81 | 1.11 | 0.9 | 6.54 | 0.85 | 0.76 | 0.94 | 0.98 | 1.1 | 0.75 | 0.97 | 0.88 | 0.69 | 0.9 | 0.93 | 0.89 |
| ZX | 0.44 | 0.16 | 1.36 | 0.16 | 1.08 | 0.59 | 1.08 | 0.46 | 0.21 | 0.59 | 0.73 | 1.03 | 0.24 | 0.6 | 0.31 | 0.24 | 0.16 | 0.6 | 0.52 |
| ZD | 1.75 | 0.94 | 1.36 | 1.53 | 1.14 | 1.53 | 11.31 | 1.53 | 1.17 | 1.33 | 1.53 | 1.16 | 1.36 | 1.55 | 1.8 | 0.82 | 3.23 | 1.53 | 1.75 |

Note:A,B,C,D,E,F,G,H,I,J,K,L,M,N,O,P,K,R,S represents *Aviceda leuphotes leuphotes, Aviceda leuphotes wolfei, Aviceda leuphotes syama, Accipiter trivirgatus indicus*, *Accipiter trivirgatus formosae, Accipiter badius cenchroides, Accipiter badius poliopsis, Ictinaetus malayensis, Spilornis cheela burmanicus, Spilornis cheela ricketti, Spilornis cheela hoya, Spilornis cheela rutherfordi, Spizaetus nipalensis nipalensis, Spizaetus nipalensis orientalis, Chrysolophus pictu, Amaurornis akool coccineipes, Streptopelia chinensis chinensis, Streptopelia chinensis formosa, Streptopelia chinensis hainana, Streptopelia chinensis tigrina* respectively.

TP,TX,TD represents the mean, minimum and maximum value of mean annual air temperature;PY,XY,DY represents the mean, minimum and maximum value of mean annual air temperature mean air temperature in January; PQ,XQ,DQ represents the mean, minimum and maximum value of mean air temperature in July ;GP,GX,GD represents the mean, minimum and maximum value of highest temperature in the warmest month; XP,XX,XD represents the mean, minimum and maximum value of lowest temperature in the coldest month; JP,JX,JD represents the mean, minimum and maximum value of sums of cumulative temperature above 0℃;SP,SX,SD represents the mean, minimum and maximum value of annual precipitation ;WP,WX,WD represents the mean, minimum and maximum value of BT;ZP,ZX, ZD represents the mean, minimum and maximum value of PER, respectively.

**Appendix 5 The membership function** parameters of different birds

|  | TP | TX | YP | YX | QP | QX | DP | DX | XP | XX | JP | JX | SP | SX | WP | WX | ZP | ZX |
| --- | --- | --- | --- | --- | --- | --- | --- | --- | --- | --- | --- | --- | --- | --- | --- | --- | --- | --- |
| A | 4 | 0.2 | 4 | 0.02 | 6 | 0.03 | 3.01796 | 3.01796 | 2.77612 | 0.00028 | 2 | 0.01 | 2 | 0.01 | 4 | 0.2 | 16 | 0.08 |
| B | 4 | 0.2 | 4 | 0.02 | 4 | 0.02 | 2.26002 | 2.26002 | 2.77612 | 0.00028 | 2 | 0.01 | 2 | 0.01 | 4 | 0.2 | 14 | 0.07 |
| C | 24 | 1.2 | 40 | 0.2 | 40 | 0.2 | 17.92878 | 17.92878 | 2.77612 | 0.00028 | 40 | 0.2 | 40 | 0.2 | 24 | 1.2 | 40 | 0.2 |
| D | 4 | 0.2 | 4 | 0.02 | 4 | 0.02 | 2.39601 | 2.39601 | 2.77612 | 0.00028 | 2 | 0.01 | 2 | 0.01 | 4 | 0.2 | 10 | 0.05 |
| E | 8 | 0.4 | 10 | 0.05 | 20 | 0.1 | 9.09905 | 9.09905 | 2.77612 | 0.00028 | 2 | 0.01 | 4 | 0.02 | 8 | 0.4 | 34 | 0.17 |
| F | 4 | 0.2 | 4 | 0.02 | 6 | 0.03 | 3.62592 | 3.62592 | 2.77612 | 0.00028 | 2 | 0.01 | 2 | 0.01 | 4 | 0.2 | 16 | 0.08 |
| G | 4 | 0.2 | 4 | 0.02 | 14 | 0.07 | 3.9189 | 3.9189 | 2.77612 | 0.00028 | 2 | 0.01 | 2 | 0.01 | 8 | 0.4 | 4 | 0.02 |
| H | 4 | 0.2 | 4 | 0.02 | 6 | 0.03 | 2.86197 | 2.86197 | 2.77612 | 0.00028 | 2 | 0.01 | 2 | 0.01 | 4 | 0.2 | 14 | 0.07 |
| i | 4 | 0.2 | 4 | 0.02 | 6 | 0.03 | 3.68892 | 3.68892 | 2.77612 | 0.00028 | 2 | 0.01 | 2 | 0.01 | 4 | 0.2 | 14 | 0.07 |
| j | 4 | 0.2 | 4 | 0.02 | 4 | 0.02 | 2.29902 | 2.29902 | 2.77612 | 0.00028 | 2 | 0.01 | 2 | 0.01 | 4 | 0.2 | 14 | 0.07 |
| K | 6 | 0.3 | 6 | 0.03 | 26 | 0.13 | 8.57784 | 8.57784 | 2.77612 | 0.00028 | 2 | 0.01 | 2 | 0.01 | 10 | 0.5 | 16 | 0.08 |
| L | 6 | 0.3 | 8 | 0.04 | 18 | 0.09 | 6.09474 | 6.09474 | 2.77612 | 0.00028 | 2 | 0.01 | 2 | 0.01 | 6 | 0.3 | 30 | 0.15 |
| M | 2 | 0.1 | 4 | 0.02 | 4 | 0.02 | 2.29502 | 2.29502 | 2.77612 | 0.00028 | 2 | 0.01 | 2 | 0.01 | 2 | 0.1 | 12 | 0.06 |
| N | 4 | 0.2 | 4 | 0.02 | 6 | 0.03 | 2.44701 | 2.44701 | 2.77612 | 0.00028 | 2 | 0.01 | 2 | 0.01 | 4 | 0.2 | 14 | 0.07 |
| O | 4 | 0.2 | 4 | 0.02 | 4 | 0.02 | 2.42501 | 2.42501 | 2.77612 | 0.00028 | 2 | 0.01 | 2 | 0.01 | 4 | 0.2 | 12 | 0.06 |
| P | 4 | 0.2 | 4 | 0.02 | 8 | 0.04 | 3.8879 | 3.8879 | 2.77612 | 0.00028 | 2 | 0.01 | 2 | 0.01 | 4 | 0.2 | 22 | 0.11 |
| Q | 4 | 0.2 | 4 | 0.02 | 4 | 0.02 | 2.499 | 2.499 | 2.77612 | 0.00028 | 2 | 0.01 | 2 | 0.01 | 4 | 0.2 | 10 | 0.05 |
| R | 6 | 0.3 | 8 | 0.04 | 24 | 0.12 | 5.80576 | 5.80576 | 2.77612 | 0.00028 | 2 | 0.01 | 2 | 0.01 | 8 | 0.4 | 14 | 0.07 |
| S | 4 | 0.2 | 4 | 0.02 | 4 | 0.02 | 2.14403 | 2.14403 | 2.77612 | 0.00028 | 2 | 0.01 | 2 | 0.01 | 4 | 0.2 | 14 | 0.07 |

Note:A,B,C,D,E,F,G,H,I,J,K,L,M,N,O,P,K,R,S represents *Aviceda leuphotes leuphotes, Aviceda leuphotes wolfei, Aviceda leuphotes syama, Accipiter trivirgatus indicus*, *Accipiter trivirgatus formosae, Accipiter badius cenchroides, Accipiter badius poliopsis, Ictinaetus malayensis, Spilornis cheela burmanicus, Spilornis cheela ricketti, Spilornis cheela hoya, Spilornis cheela rutherfordi, Spizaetus nipalensis nipalensis, Spizaetus nipalensis orientalis, Chrysolophus pictu, Amaurornis akool coccineipes, Streptopelia chinensis chinensis, Streptopelia chinensis formosa, Streptopelia chinensis hainana, Streptopelia chinensis tigrina* respectively.

TP,TX represents α,β value of mean annual air temperature;YP,YX represents α, β value of mean air temperature in January; QP,QX represents α,β value of mean air temperature in July ; DP,DX represents α,β value of highest temperature in the warmest month; XP,XX represents α,β value of lowest temperature in the coldest month; JP,JX represents α,β value of sums of cumulative temperature above 0℃;SP,SX represents α,β value of annual precipitation ;WP,WX represents α,β value of BT;ZP,ZX represents α,β value of PER respectively for the membership function parameters.
