# Supplementary material for: Can changes in the distributions of resident birds in China over the past 50 years be attributed to climate change?
Source: Ecol Evol. 2015 May 11;5(11):2215–33. doi: 10.1002/ece3.1513 (PMC4461423; doi:10.1002/ece3.1513)
Supplement: Supplementary file 4 [file ece30005-2215-sd4.doc]

**Appendix S6. The weight coefficient of climatic factors**

| year | tp | yp | qp | zd | zx | jw | js | sw | gz | year | tp | yp | qp | zd | zx | jw | js | sw | gz |
| --- | --- | --- | --- | --- | --- | --- | --- | --- | --- | --- | --- | --- | --- | --- | --- | --- | --- | --- | --- |
| 1961 | 0.147 | 0.102 | 0.232 | 0.272 | 0.091 | 0.005 | 0.131 | 0.005 | 0.014 | 1986 | 0.147 | 0.105 | 0.227 | 0.273 | 0.094 | 0.005 | 0.132 | 0.005 | 0.013 |
| 1962 | 0.147 | 0.103 | 0.231 | 0.269 | 0.094 | 0.005 | 0.133 | 0.005 | 0.013 | 1987 | 0.149 | 0.107 | 0.228 | 0.271 | 0.093 | 0.005 | 0.133 | 0.005 | 0.010 |
| 1963 | 0.149 | 0.102 | 0.230 | 0.269 | 0.094 | 0.005 | 0.133 | 0.005 | 0.013 | 1988 | 0.146 | 0.105 | 0.230 | 0.275 | 0.093 | 0.005 | 0.130 | 0.005 | 0.010 |
| 1964 | 0.147 | 0.105 | 0.228 | 0.272 | 0.093 | 0.005 | 0.134 | 0.005 | 0.011 | 1989 | 0.148 | 0.105 | 0.225 | 0.275 | 0.094 | 0.005 | 0.132 | 0.005 | 0.011 |
| 1965 | 0.148 | 0.107 | 0.228 | 0.268 | 0.093 | 0.005 | 0.134 | 0.005 | 0.012 | 1990 | 0.150 | 0.106 | 0.228 | 0.269 | 0.094 | 0.005 | 0.133 | 0.005 | 0.011 |
| 1966 | 0.148 | 0.105 | 0.227 | 0.274 | 0.091 | 0.005 | 0.133 | 0.005 | 0.011 | 1991 | 0.148 | 0.105 | 0.231 | 0.272 | 0.091 | 0.005 | 0.132 | 0.005 | 0.011 |
| 1967 | 0.147 | 0.103 | 0.231 | 0.271 | 0.091 | 0.005 | 0.134 | 0.005 | 0.013 | 1992 | 0.148 | 0.106 | 0.225 | 0.271 | 0.097 | 0.005 | 0.133 | 0.005 | 0.010 |
| 1968 | 0.148 | 0.104 | 0.229 | 0.271 | 0.092 | 0.005 | 0.133 | 0.005 | 0.013 | 1993 | 0.150 | 0.105 | 0.228 | 0.265 | 0.095 | 0.005 | 0.135 | 0.005 | 0.010 |
| 1969 | 0.147 | 0.103 | 0.229 | 0.273 | 0.092 | 0.005 | 0.134 | 0.005 | 0.012 | 1994 | 0.147 | 0.104 | 0.231 | 0.274 | 0.093 | 0.005 | 0.129 | 0.005 | 0.012 |
| 1970 | 0.147 | 0.104 | 0.229 | 0.271 | 0.092 | 0.005 | 0.134 | 0.005 | 0.012 | 1995 | 0.148 | 0.104 | 0.227 | 0.275 | 0.096 | 0.005 | 0.131 | 0.005 | 0.010 |
| 1971 | 0.147 | 0.104 | 0.230 | 0.271 | 0.094 | 0.005 | 0.133 | 0.005 | 0.011 | 1996 | 0.149 | 0.105 | 0.229 | 0.269 | 0.095 | 0.005 | 0.133 | 0.005 | 0.011 |
| 1972 | 0.146 | 0.106 | 0.225 | 0.277 | 0.093 | 0.005 | 0.132 | 0.005 | 0.011 | 1997 | 0.146 | 0.104 | 0.229 | 0.278 | 0.093 | 0.005 | 0.129 | 0.005 | 0.012 |
| 1973 | 0.148 | 0.105 | 0.228 | 0.271 | 0.094 | 0.005 | 0.132 | 0.005 | 0.012 | 1998 | 0.151 | 0.103 | 0.232 | 0.271 | 0.092 | 0.005 | 0.132 | 0.005 | 0.011 |
| 1974 | 0.147 | 0.105 | 0.231 | 0.268 | 0.093 | 0.005 | 0.133 | 0.005 | 0.013 | 1999 | 0.147 | 0.105 | 0.227 | 0.280 | 0.093 | 0.005 | 0.128 | 0.005 | 0.011 |
| 1975 | 0.148 | 0.106 | 0.226 | 0.272 | 0.093 | 0.005 | 0.133 | 0.005 | 0.012 | 2000 | 0.144 | 0.101 | 0.233 | 0.283 | 0.091 | 0.005 | 0.128 | 0.005 | 0.010 |
| 1976 | 0.148 | 0.107 | 0.224 | 0.270 | 0.093 | 0.005 | 0.135 | 0.005 | 0.012 | 2001 | 0.146 | 0.102 | 0.233 | 0.279 | 0.090 | 0.005 | 0.128 | 0.005 | 0.012 |
| 1977 | 0.148 | 0.100 | 0.230 | 0.277 | 0.090 | 0.005 | 0.133 | 0.005 | 0.012 | 2002 | 0.148 | 0.106 | 0.229 | 0.278 | 0.091 | 0.005 | 0.129 | 0.005 | 0.010 |
| 1978 | 0.148 | 0.102 | 0.231 | 0.274 | 0.091 | 0.005 | 0.132 | 0.005 | 0.013 | 2003 | 0.150 | 0.106 | 0.228 | 0.271 | 0.094 | 0.005 | 0.132 | 0.005 | 0.010 |
| 1979 | 0.149 | 0.107 | 0.224 | 0.271 | 0.094 | 0.005 | 0.133 | 0.005 | 0.012 | 2004 | 0.148 | 0.104 | 0.227 | 0.278 | 0.092 | 0.005 | 0.129 | 0.005 | 0.012 |
| 1980 | 0.147 | 0.104 | 0.229 | 0.273 | 0.092 | 0.005 | 0.133 | 0.005 | 0.013 | 2005 | 0.147 | 0.103 | 0.231 | 0.279 | 0.093 | 0.005 | 0.129 | 0.005 | 0.009 |
| 1981 | 0.146 | 0.103 | 0.232 | 0.276 | 0.092 | 0.005 | 0.132 | 0.005 | 0.009 | 2006 | 0.148 | 0.103 | 0.231 | 0.279 | 0.091 | 0.005 | 0.128 | 0.005 | 0.010 |
| 1982 | 0.148 | 0.107 | 0.227 | 0.270 | 0.095 | 0.005 | 0.132 | 0.005 | 0.011 | 2007 | 0.150 | 0.105 | 0.228 | 0.273 | 0.096 | 0.005 | 0.129 | 0.005 | 0.010 |
| 1983 | 0.148 | 0.105 | 0.225 | 0.273 | 0.093 | 0.005 | 0.134 | 0.005 | 0.011 | 2008 | 0.149 | 0.102 | 0.233 | 0.274 | 0.090 | 0.005 | 0.131 | 0.005 | 0.011 |
| 1984 | 0.149 | 0.104 | 0.228 | 0.269 | 0.093 | 0.005 | 0.135 | 0.005 | 0.012 | 2009 | 0.149 | 0.105 | 0.230 | 0.272 | 0.093 | 0.005 | 0.129 | 0.005 | 0.013 |
| 1985 | 0.148 | 0.104 | 0.228 | 0.269 | 0.094 | 0.005 | 0.134 | 0.005 | 0.014 | 2010 | 0.144 | 0.102 | 0.231 | 0.289 | 0.089 | 0.004 | 0.126 | 0.004 | 0.009 |

Note :tp,yp,qp,zd,zx,jw,js,sw and gz represents mean annual air temperature, mean air temperature in January, mean air temperature in July , the highest temperature in warmest month , the lowest temperature in coldest month , sums of cumulative temperature above 0℃, annual precipitation ,BT and PER respectively.

**Appendix S7.Model performance by Kappa statistic for different bird species or subspecies**

| **Species or subspecies of birds** | Kappa(k) | Degree of agreement |
| --- | --- | --- |
| *Aviceda leuphotes leuphotes* | 0.69 | good |
| *Aviceda leuphotes wolfei* | 0.60 | good |
| *Aviceda leuphotes syama* | 0.54 | fair |
| *Accipiter trivirgatus indicus* | 0.84 | very good |
| *Accipiter trivirgatus formosae* | 0.67 | good |
| *Accipiter badius cenchroides* | 0.79 | very good |
| *Accipiter badius poliopsis* | 0.65 | good |
| *Ictinaetus malayensis* | 0.75 | very good |
| *Spilornis cheela burmanicus* | 0.67 | good |
| *Spilornis cheela ricketti* | 0.65 | good |
| *Spilornis cheela hoya* | 0.60 | good |
| *Spilornis cheela rutherfordi* | 0.75 | very good |
| *Spizaetus nipalensis nipalensis* | 0.64 | good |
| *Spizaetus nipalensis orientalis* | 0.69 | good |
| *Chrysolophus pictu* | 0.74 | very good |
| *Amaurornis akool coccineipes* | 0.71 | very good |
| *Streptopelia chinensis chinensis* | 0.75 | very good |
| *Streptopelia chinensis formosa* | 0.68 | good |
| *Streptopelia chinensis hainana* | 0.53 | fair |
| *Streptopelia chinensis tigrina* | 0.60 | good |

Notes: the k is calculated as follows

$k=\left[\left(a+d\right)-\left(\left(\left(a+c\right)\left(a+b\right)+\left(b+d\right)\left(c+d\right)\right)\div N\right)\right]\div\left[N-\left(\left(\left(a+c\right)\left(a+b\right)+\left(b+d\right)\left(c+d\right)\right)\div N\right)\right]$where

$a=$the number of cases predicted present when actually present

$b=$the number of cases predicted present when actually absent

$c=$the number of cases predicted absent when actually present

$d=$the number of cases predicted absent when actually absent

$$N=a+b+c+d$$

Using the ranges of agreement for the kappa statistic proposed by Monserud & Leemans(1992) to describe the degree of agreement.

Monserud ,R.A, R.Leemans.1992. Comparing global vegetation maps with the Kappa statistic.Ecological Modelling.62 :275-293

**Appendix S8. The degree of grey incidence of observed changes at the southern and northern boundaries of distributions of birds with different climatic factors**

|  | T1 | T2 | T3 | T4 | T5 | T6 | T7 | T8 | T9 | T1 | T2 | T3 | T4 | T5 | T6 | T7 | T8 | T9 |
| --- | --- | --- | --- | --- | --- | --- | --- | --- | --- | --- | --- | --- | --- | --- | --- | --- | --- | --- |
|  | Southern boundary | | | | | | | | | Northern boundary | | | | | | | | |
| ALL | 0.64 | 0.57 | 0.70 | 0.66 | 0.56 | 0.72 | 0.61 | 0.72 | 0.61 | 0.91 | 0.83 | 0.83 | 0.93 | 0.78 | 0.84 | 0.59 | 0.84 | 0.48 |
| ALW | 0.43 | 0.48 | 0.40 | 0.41 | 0.49 | 0.39 | 0.62 | 0.39 | 0.60 | 0.56 | 0.51 | 0.64 | 0.56 | 0.50 | 0.62 | 0.62 | 0.62 | 0.68 |
| ALS | 0.64 | 0.57 | 0.70 | 0.66 | 0.56 | 0.72 | 0.61 | 0.72 | 0.61 | 0.64 | 0.57 | 0.70 | 0.66 | 0.56 | 0.72 | 0.61 | 0.72 | 0.61 |
| ATI | 0.64 | 0.57 | 0.70 | 0.66 | 0.56 | 0.72 | 0.61 | 0.72 | 0.61 | 0.78 | 0.80 | 0.77 | 0.75 | 0.90 | 0.78 | 0.75 | 0.78 | 0.39 |
| ATF | 0.64 | 0.57 | 0.70 | 0.66 | 0.56 | 0.72 | 0.61 | 0.72 | 0.61 | 0.77 | 0.70 | 0.83 | 0.79 | 0.66 | 0.86 | 0.56 | 0.85 | 0.59 |
| ABC | 0.64 | 0.57 | 0.70 | 0.66 | 0.56 | 0.72 | 0.61 | 0.72 | 0.61 | 0.67 | 0.72 | 0.62 | 0.66 | 0.77 | 0.63 | 0.68 | 0.63 | 0.44 |
| ABP | 0.64 | 0.57 | 0.70 | 0.66 | 0.56 | 0.72 | 0.61 | 0.72 | 0.61 | 0.80 | 0.74 | 0.84 | 0.80 | 0.79 | 0.85 | 0.69 | 0.85 | 0.51 |
| IM | 0.64 | 0.57 | 0.70 | 0.66 | 0.56 | 0.72 | 0.61 | 0.72 | 0.61 | 0.80 | 0.74 | 0.84 | 0.80 | 0.79 | 0.85 | 0.69 | 0.85 | 0.51 |
| SCB | 0.51 | 0.43 | 0.60 | 0.52 | 0.42 | 0.60 | 0.54 | 0.60 | 0.75 | 0.82 | 0.87 | 0.76 | 0.83 | 0.75 | 0.79 | 0.53 | 0.79 | 0.50 |
| SCR | 0.64 | 0.57 | 0.70 | 0.66 | 0.56 | 0.72 | 0.61 | 0.72 | 0.61 | 0.75 | 0.75 | 0.75 | 0.73 | 0.83 | 0.76 | 0.82 | 0.76 | 0.38 |
| SCH | 0.64 | 0.57 | 0.70 | 0.66 | 0.56 | 0.72 | 0.61 | 0.72 | 0.61 | 0.64 | 0.57 | 0.70 | 0.66 | 0.56 | 0.72 | 0.61 | 0.72 | 0.61 |
| SCRT | 0.64 | 0.57 | 0.70 | 0.66 | 0.56 | 0.72 | 0.61 | 0.72 | 0.61 | 0.64 | 0.57 | 0.70 | 0.66 | 0.56 | 0.72 | 0.61 | 0.72 | 0.61 |
| SNN | 0.51 | 0.43 | 0.60 | 0.52 | 0.42 | 0.60 | 0.54 | 0.60 | 0.75 | 0.83 | 0.86 | 0.81 | 0.79 | 0.82 | 0.81 | 0.69 | 0.81 | 0.41 |
| SNO | 0.40 | 0.45 | 0.38 | 0.39 | 0.54 | 0.37 | 0.67 | 0.37 | 0.59 | 0.64 | 0.57 | 0.70 | 0.66 | 0.56 | 0.72 | 0.61 | 0.72 | 0.61 |
| CP | 0.55 | 0.46 | 0.60 | 0.57 | 0.45 | 0.60 | 0.52 | 0.60 | 0.72 | 0.80 | 0.74 | 0.84 | 0.80 | 0.79 | 0.85 | 0.69 | 0.85 | 0.51 |
| AAC | 0.40 | 0.45 | 0.38 | 0.39 | 0.54 | 0.37 | 0.67 | 0.37 | 0.59 | 0.77 | 0.70 | 0.83 | 0.79 | 0.66 | 0.86 | 0.56 | 0.85 | 0.59 |
| SCC | 0.64 | 0.57 | 0.70 | 0.66 | 0.56 | 0.72 | 0.61 | 0.72 | 0.61 | 0.67 | 0.71 | 0.62 | 0.66 | 0.77 | 0.63 | 0.68 | 0.63 | 0.43 |
| SCF | 0.77 | 0.70 | 0.83 | 0.79 | 0.66 | 0.86 | 0.56 | 0.85 | 0.59 | 0.77 | 0.70 | 0.83 | 0.79 | 0.66 | 0.86 | 0.56 | 0.85 | 0.59 |
| SCH | 0.64 | 0.57 | 0.70 | 0.66 | 0.56 | 0.72 | 0.61 | 0.72 | 0.61 | 0.66 | 0.60 | 0.71 | 0.67 | 0.69 | 0.72 | 0.75 | 0.72 | 0.53 |
| SCT | 0.40 | 0.45 | 0.38 | 0.39 | 0.54 | 0.37 | 0.67 | 0.37 | 0.59 | 0.64 | 0.57 | 0.70 | 0.66 | 0.56 | 0.72 | 0.61 | 0.72 | 0.61 |

Note:T1,T2,T3,T4,T5,T6,T7,T8,T9 represents mean annual air temperature, mean air temperature in January, mean air temperature in July , the highest temperature in warmest month , the lowest temperature in coldest month , sums of cumulative temperature above 0℃, annual precipitation ,BT and PER respectively.

ALL(*Aviceda leuphotes leuphotes)*,ALW*(Aviceda leuphotes wolfei),* ALS*(Aviceda leuphotes syama*), ATI(*Accipiter trivirgatus indicus)*, ATF(*Accipiter trivirgatus formosae),*ABC(*Accipiter badius cenchroides),*ABP*(Accipiter badius poliopsis*), IM(*Ictinaetus malayensis*),SCB(*Spilornis cheela burmanicus)*,SCR*(Spilornis cheela ricketti),* SCH*(Spilornis cheela hoya),SCRT(Spilornis cheela rutherfordi)*,SNN(*Spizaetus nipalensis nipalensis),SNO(Spizaetus nipalensis orientalis*), CP(*Chrysolophus pictu*),AAC(*Amaurornis akool coccineipes*),SCC(*Streptopelia chinensis chinensis),,SCF(Streptopelia chinensis formosa*),*SCH(Streptopelia chinensis hainana)*,SCT(*Streptopelia chinensis tigrina*),

Same Appendix S6 and S7.

**Appendix S9. The degree of grey incidence of observed changes at the eastern and western boundaries of bird distributions with climatic factors**

|  | T1 | T2 | T3 | T4 | T5 | T6 | T7 | T8 | T9 | T1 | T2 | T3 | T4 | T5 | T6 | T7 | T8 | T9 |
| --- | --- | --- | --- | --- | --- | --- | --- | --- | --- | --- | --- | --- | --- | --- | --- | --- | --- | --- |
|  | Western boundary | | | | | | | | | Eastern boundary | | | | | | | | |
| ALL | 0.64 | 0.57 | 0.70 | 0.66 | 0.56 | 0.72 | 0.61 | 0.72 | 0.61 | 0.73 | 0.81 | 0.66 | 0.71 | 0.86 | 0.67 | 0.62 | 0.67 | 0.49 |
| ALW | 0.43 | 0.48 | 0.41 | 0.42 | 0.49 | 0.39 | 0.61 | 0.40 | 0.60 | 0.51 | 0.43 | 0.60 | 0.52 | 0.42 | 0.60 | 0.54 | 0.60 | 0.75 |
| ALS | 0.64 | 0.57 | 0.70 | 0.66 | 0.56 | 0.72 | 0.61 | 0.72 | 0.61 | 0.64 | 0.57 | 0.70 | 0.66 | 0.56 | 0.72 | 0.61 | 0.72 | 0.61 |
| ATI | 0.40 | 0.45 | 0.38 | 0.39 | 0.54 | 0.37 | 0.67 | 0.37 | 0.59 | 0.77 | 0.86 | 0.70 | 0.75 | 0.82 | 0.70 | 0.61 | 0.70 | 0.45 |
| ATF | 0.40 | 0.45 | 0.38 | 0.39 | 0.54 | 0.37 | 0.67 | 0.37 | 0.59 | 0.64 | 0.57 | 0.70 | 0.66 | 0.56 | 0.72 | 0.61 | 0.72 | 0.61 |
| ABC | 0.64 | 0.57 | 0.70 | 0.66 | 0.56 | 0.72 | 0.61 | 0.72 | 0.61 | 0.64 | 0.57 | 0.70 | 0.66 | 0.56 | 0.72 | 0.61 | 0.72 | 0.61 |
| ABP | 0.64 | 0.57 | 0.70 | 0.66 | 0.56 | 0.72 | 0.61 | 0.72 | 0.61 | 0.80 | 0.74 | 0.84 | 0.80 | 0.79 | 0.85 | 0.69 | 0.85 | 0.51 |
| IM | 0.48 | 0.44 | 0.43 | 0.46 | 0.40 | 0.44 | 0.43 | 0.44 | 0.73 | 0.77 | 0.70 | 0.83 | 0.79 | 0.66 | 0.86 | 0.56 | 0.85 | 0.59 |
| SCB | 0.51 | 0.43 | 0.60 | 0.52 | 0.42 | 0.60 | 0.54 | 0.60 | 0.75 | 0.82 | 0.87 | 0.76 | 0.83 | 0.75 | 0.79 | 0.53 | 0.80 | 0.50 |
| SCR | 0.64 | 0.57 | 0.70 | 0.66 | 0.56 | 0.72 | 0.61 | 0.72 | 0.61 | 0.64 | 0.57 | 0.70 | 0.66 | 0.56 | 0.72 | 0.61 | 0.72 | 0.61 |
| SCH | 0.64 | 0.57 | 0.70 | 0.66 | 0.56 | 0.72 | 0.61 | 0.72 | 0.61 | 0.64 | 0.57 | 0.70 | 0.66 | 0.56 | 0.72 | 0.61 | 0.72 | 0.61 |
| SCRT | 0.64 | 0.57 | 0.70 | 0.66 | 0.56 | 0.72 | 0.61 | 0.72 | 0.61 | 0.64 | 0.57 | 0.70 | 0.66 | 0.56 | 0.72 | 0.61 | 0.72 | 0.61 |
| SNN | 0.64 | 0.57 | 0.70 | 0.66 | 0.56 | 0.72 | 0.61 | 0.72 | 0.61 | 0.75 | 0.75 | 0.75 | 0.73 | 0.83 | 0.76 | 0.82 | 0.76 | 0.38 |
| SNO | 0.40 | 0.45 | 0.38 | 0.39 | 0.54 | 0.37 | 0.67 | 0.37 | 0.59 | 0.64 | 0.57 | 0.70 | 0.66 | 0.56 | 0.72 | 0.61 | 0.72 | 0.61 |
| CP | 0.44 | 0.47 | 0.41 | 0.43 | 0.43 | 0.41 | 0.45 | 0.41 | 0.71 | 0.79 | 0.84 | 0.70 | 0.77 | 0.85 | 0.72 | 0.63 | 0.72 | 0.46 |
| AAC | 0.40 | 0.45 | 0.38 | 0.39 | 0.54 | 0.37 | 0.67 | 0.37 | 0.59 | 0.84 | 0.74 | 0.85 | 0.86 | 0.68 | 0.89 | 0.58 | 0.88 | 0.56 |
| SCC | 0.64 | 0.57 | 0.70 | 0.66 | 0.56 | 0.72 | 0.61 | 0.72 | 0.61 | 0.82 | 0.81 | 0.72 | 0.79 | 0.84 | 0.73 | 0.72 | 0.74 | 0.40 |
| SCF | 0.77 | 0.70 | 0.83 | 0.79 | 0.66 | 0.86 | 0.56 | 0.85 | 0.59 | 0.77 | 0.70 | 0.83 | 0.79 | 0.66 | 0.86 | 0.56 | 0.85 | 0.59 |
| SCH | 0.64 | 0.57 | 0.70 | 0.66 | 0.56 | 0.72 | 0.61 | 0.72 | 0.61 | 0.66 | 0.60 | 0.71 | 0.67 | 0.69 | 0.72 | 0.75 | 0.72 | 0.53 |
| SCT | 0.64 | 0.57 | 0.70 | 0.66 | 0.56 | 0.72 | 0.61 | 0.72 | 0.61 | 0.64 | 0.57 | 0.70 | 0.66 | 0.56 | 0.72 | 0.61 | 0.72 | 0.61 |

**Appendix S10. The degree of grey incidence of observed changes in latitude and longitude of distribution centers of the birds with climatic factors**

|  | T1 | T2 | T3 | T4 | T5 | T6 | T7 | T8 | T9 | T1 | T2 | T3 | T4 | T5 | T6 | T7 | T8 | T9 |
| --- | --- | --- | --- | --- | --- | --- | --- | --- | --- | --- | --- | --- | --- | --- | --- | --- | --- | --- |
|  | longitude of distribution center | | | | | | | | | latitude of distribution center | | | | | | | | |
| ALL | 0.81 | 0.86 | 0.72 | 0.80 | 0.94 | 0.73 | 0.68 | 0.73 | 0.41 | 0.84 | 0.91 | 0.74 | 0.82 | 0.87 | 0.75 | 0.62 | 0.75 | 0.43 |
| ALW | 0.52 | 0.56 | 0.49 | 0.50 | 0.53 | 0.47 | 0.54 | 0.47 | 0.69 | 0.56 | 0.62 | 0.50 | 0.53 | 0.55 | 0.49 | 0.55 | 0.49 | 0.63 |
| ALS | 0.64 | 0.57 | 0.70 | 0.66 | 0.56 | 0.72 | 0.61 | 0.72 | 0.61 | 0.64 | 0.57 | 0.70 | 0.66 | 0.56 | 0.72 | 0.61 | 0.72 | 0.61 |
| ATI | 0.87 | 0.97 | 0.77 | 0.84 | 0.81 | 0.77 | 0.57 | 0.77 | 0.46 | 0.82 | 0.72 | 0.92 | 0.86 | 0.64 | 0.90 | 0.63 | 0.90 | 0.52 |
| ATF | 0.40 | 0.45 | 0.38 | 0.39 | 0.54 | 0.37 | 0.67 | 0.37 | 0.59 | 0.77 | 0.70 | 0.83 | 0.79 | 0.66 | 0.86 | 0.56 | 0.85 | 0.59 |
| ABC | 0.51 | 0.59 | 0.49 | 0.51 | 0.52 | 0.51 | 0.51 | 0.50 | 0.67 | 0.86 | 0.83 | 0.77 | 0.85 | 0.73 | 0.80 | 0.51 | 0.80 | 0.54 |
| ABP | 0.84 | 0.79 | 0.88 | 0.84 | 0.72 | 0.89 | 0.62 | 0.89 | 0.53 | 0.88 | 0.80 | 0.91 | 0.87 | 0.71 | 0.91 | 0.61 | 0.91 | 0.54 |
| IM | 0.48 | 0.51 | 0.46 | 0.47 | 0.47 | 0.46 | 0.46 | 0.46 | 0.70 | 0.91 | 0.84 | 0.80 | 0.90 | 0.81 | 0.81 | 0.60 | 0.81 | 0.47 |
| SCB | 0.54 | 0.62 | 0.52 | 0.53 | 0.71 | 0.49 | 0.78 | 0.50 | 0.46 | 0.52 | 0.59 | 0.52 | 0.51 | 0.69 | 0.49 | 0.73 | 0.49 | 0.53 |
| SCR | 0.42 | 0.39 | 0.46 | 0.45 | 0.37 | 0.47 | 0.41 | 0.47 | 0.81 | 0.75 | 0.75 | 0.75 | 0.73 | 0.83 | 0.76 | 0.82 | 0.76 | 0.38 |
| SCH | 0.64 | 0.57 | 0.70 | 0.66 | 0.56 | 0.72 | 0.61 | 0.72 | 0.61 | 0.64 | 0.57 | 0.70 | 0.66 | 0.56 | 0.72 | 0.61 | 0.72 | 0.61 |
| SCRT | 0.64 | 0.57 | 0.70 | 0.66 | 0.56 | 0.72 | 0.61 | 0.72 | 0.61 | 0.64 | 0.57 | 0.70 | 0.66 | 0.56 | 0.72 | 0.61 | 0.72 | 0.61 |
| SNN | 0.55 | 0.52 | 0.60 | 0.59 | 0.48 | 0.62 | 0.40 | 0.61 | 0.79 | 0.68 | 0.68 | 0.71 | 0.65 | 0.76 | 0.69 | 0.81 | 0.70 | 0.40 |
| SNO | 0.40 | 0.45 | 0.38 | 0.39 | 0.54 | 0.37 | 0.67 | 0.37 | 0.59 | 0.40 | 0.45 | 0.38 | 0.39 | 0.54 | 0.37 | 0.67 | 0.37 | 0.59 |
| CP | 0.69 | 0.75 | 0.63 | 0.67 | 0.82 | 0.64 | 0.63 | 0.64 | 0.45 | 0.65 | 0.71 | 0.59 | 0.63 | 0.67 | 0.59 | 0.64 | 0.59 | 0.50 |
| AAC | 0.63 | 0.63 | 0.67 | 0.60 | 0.75 | 0.65 | 0.89 | 0.65 | 0.42 | 0.83 | 0.80 | 0.86 | 0.79 | 0.69 | 0.82 | 0.68 | 0.82 | 0.45 |
| SCC | 0.86 | 0.89 | 0.81 | 0.82 | 0.80 | 0.82 | 0.66 | 0.82 | 0.42 | 0.58 | 0.63 | 0.53 | 0.57 | 0.77 | 0.52 | 0.76 | 0.52 | 0.45 |
| SCF | 0.40 | 0.45 | 0.38 | 0.39 | 0.54 | 0.37 | 0.67 | 0.37 | 0.59 | 0.40 | 0.45 | 0.38 | 0.39 | 0.54 | 0.37 | 0.67 | 0.37 | 0.59 |
| SCH | 0.66 | 0.60 | 0.71 | 0.67 | 0.69 | 0.72 | 0.75 | 0.72 | 0.53 | 0.66 | 0.60 | 0.71 | 0.67 | 0.69 | 0.72 | 0.75 | 0.72 | 0.53 |
| SCT | 0.77 | 0.70 | 0.83 | 0.79 | 0.66 | 0.86 | 0.56 | 0.85 | 0.59 | 0.40 | 0.45 | 0.38 | 0.39 | 0.54 | 0.37 | 0.67 | 0.37 | 0.59 |
